# High yield bacterial expression, purification and characterisation of bioactive Human Tousled-like Kinase 1B involved in cancer

**DOI:** 10.1038/s41598-018-22744-5

**Published:** 2018-03-19

**Authors:** Siddhant Bhoir, Althaf Shaik, Vijay Thiruvenkatam, Sivapriya Kirubakaran

**Affiliations:** 1 Dicipline of Biological Engineering, Indian Institute of Technology Gandhinagar, Simkheda, Palaj, Gandhinagar, 382355 Gujarat India; 2 Dicipline of Chemistry, Indian Institute of Technology Gandhinagar, Simkheda, Palaj, Gandhinagar, 382355 Gujarat India; 3 Dicipline of Physics, Indian Institute of Technology Gandhinagar, Simkheda, Palaj, Gandhinagar, 382355 Gujarat India

## Abstract

Human Tousled-like kinases (TLKs) are highly conserved serine/threonine protein kinases responsible for cell proliferation, DNA repair, and genome surveillance. Their possible involvement in cancer via efficient DNA repair mechanisms have made them clinically relevant molecular targets for anticancer therapy. Innovative approaches in chemical biology have played a key role in validating the importance of kinases as molecular targets. However, the detailed understanding of the protein structure and the mechanisms of protein–drug interaction through biochemical and biophysical techniques demands a method for the production of an active protein of exceptional stability and purity on a large scale. We have designed a bacterial expression system to express and purify biologically active, wild-type Human Tousled-like Kinase 1B (hTLK1B) by co-expression with the protein phosphatase from bacteriophage λ. We have obtained remarkably high amounts of the soluble and homogeneously dephosphorylated form of biologically active hTLK1B with our unique, custom-built vector design strategy. The recombinant hTLK1B can be used for the structural studies and may further facilitate the development of new TLK inhibitors for anti-cancer therapy using a structure-based drug design approach.

## Introduction

Cancer is considered to be a severe health concern worldwide^1^. Deregulation of the kinase activity has emerged as a primary mechanism by which cancer cells evade normal physiological constraints on the growth and survival. Such aberrant functions of the kinases in a cancer cell have highlighted them as one of the most successful families of drug targets^2^. The Tousled-Like Kinases (TLK1 and TLK2) are highly conserved serine/threonine protein kinases involved in DNA repair, chromatin assembly, transcription and chromosome segregation^3^. Their discernible role in the genome surveillance, and in cancer development via efficient DNA repair mechanisms has been well substantiated^3^. Human TLKs are frequently overexpressed in breast cancer, prostate cancer and cholangiocarcinoma, and often correspond to reduced sensitivity towards chemo- and radiomimetic therapies. In response to genotoxic stress, TLK1B, a splice variant of TLK1, becomes translationally upregulated and interacts specifically with Asf1 and Rad9, and promotes chromatin-remodelling coupled DNA repair at the double-strand break (DSB) ends^3^. The mutations in the DNA repair genes and the checkpoint functions in most cancers makes them overly dependent on such alternative pathways for their survival. As high TLK1B expression is thought to be a critical transition during tumorigenesis, pre-emptive inhibition of these kinases by the specific inhibitors could choke a crucial step in the formation of tumours^4^. ATM and ATR act as central effectors of the DNA damage relay and constitute a backbone of the DNA damage response (DDR)^5^. Whereas other protein kinases including ATM, CDKs, PLK1, AKT, Nek1 (Never in Mitosis A-related kinase 1)^6^ and relevant to this article, TLK1^7^ modulate the strength of the response. Nek1, as a target of TLK1/1B, was recently uncovered through a novel proteomic screen thereby opening up a new avenue for a possible role of TLKs in dealing with the damage^8^. The development of new protein kinase inhibitors requires an in-depth knowledge of kinase regulation, its activity and its ability to bind to the drugs^9^. However, the detailed understanding of the protein structure and the mechanisms of protein–drug interaction through biochemical and biophysical techniques demands a method for the production of an active protein of exceptional stability and purity on a large scale^10^. The most common method utilised for the expression of the active forms of Human Tousled-like kinases (both TLK1 and TLK2) is from the insect cell culture^11^ or with minimal yields in bacteria^12^. Whereas the insect cell culture can provide milligram amounts of protein, the process demands time and cost. Many kinases suffer from poor solubility, incorrect folding or aggregation when expressed in prokaryotic hosts and besides, can undergo heterogeneous auto-phosphorylation^13^. One of the earlier approaches to obtaining homogeneous protein is to phosphorylate or dephosphorylate the kinase *in vitro* and then purify a particular phosphoform by chromatography. However, this method is technically challenging and yields a moderate amount of protein. An alternative method is to express a phosphatase along with the target kinase so that a high yield of non-phosphorylated kinase protein can be produced without the need for subsequent treatment with phosphatase. *Seeliger et al. (2005)* and *Kristelly et al. (2011)* have successfully produced active soluble Src family kinases in *Escherichia coli* by expressing it along with the tyrosine phosphatase YopH^10,14^. Moreover, *Wang et al. (2008)* also implemented this method to produce homogenous non-phosphorylated BTK protein from insect cells by the same approach without a reduction in yield or solubility^15^. Likewise, *Parton et al. (2016)* created a library of His-tagged human kinase domain constructs to determine their expression levels in a simple automated bacterial expression system where phosphatase co-expression (YopH for Tyr kinases and lambda for Ser/Thr kinases) was used^16^. The beneficial effects of co-expressing the protein of interest along with the bacteriophage λ phosphatase have been previously described by *Garrote et al*. *(2014)* to express, purify and crystallise TLK2-Kinase domain^17^. However, to the best of our knowledge, there are no reports which demonstrate the homogeneous, unphosphorylated, soluble expression of the wild-type hTLK1B, in a prokaryotic organism such as *E. coli* using a co-expression strategy. Here, we report a method for bacterial expression and purification of the wild-type hTLK1B by co-expressing it along with bacteriophage lambda protein phosphatase.

## Materials and Methods

### Materials

pETDUET-1^™^ DNA (Cat. 71146-3) vector was obtained from Novagen (Merck Biosciences Division, Darmstadt, Germany). The full-length, 1647 bp long, wildtype Human TLK1B gene was obtained through a custom gene synthesis service from GeneScript Biotech Corporation, Nanjing, China. High-Fidelity (HF^®^) Restriction Endonucleases (BamHI, EcoRI, MfeI and XhoI), CutSmart^®^ Buffer, T4 DNA Ligase and T4 DNA Ligase Reaction Buffer for the cloning procedures were purchased from New England Biolabs (Ipswich, MA, USA). Plasmid DNA Purification Kit and QIAquick^®^ Gel Extraction Kit were purchased from Qiagen (Hilden, Germany). *Escherichia coli* DH5α^™^ (Cat. 18265-017) competent cells were purchased from Invitrogen Corporation (Carlsbad, CA, USA) and Rosetta Gami 2^™^ (DE3) pLysS (Cat. 71352) competent cells were purchased from Novagen (Merck Biosciences Division, Darmstadt, Germany). Ampicillin, Chloramphenicol, Isopropyl-β-D-1-thiogalactopyranoside (IPTG), Luria-Bertani (LB) broth, Luria-Bertani (LB) agar, Trizma^®^ (Tris base), Sodium Chloride (NaCl), Glycerol, Triton-X l00, Tween-20, Imidazole, Tris (2-Carboxyethyl) Phosphine Hydrochloride (TCEP), Acrylamide, N,N′-Methylenebisacrylamide, Tetramethylethylenediamine (TEMED), Magnesium chloride, Ammonium Per Sulphate (APS), Sodium Dodecyl Sulphate (SDS), β-Mercaptoethanol (BME), Bromophenol Blue, Coomassie Brilliant Blue R-250, Non-Fat Milk, Ponceau S, Bovine Serum Albumin (BSA) and Nuclease-Free water were purchased from Sigma-Aldrich (Darmstadt, Germany). Complete^™^ EDTA-Free Protease Inhibitor tablets (Cat. 04693132001) were obtained from Roche (Basel, Switzerland). Nuvia^™^ Immobilized Metal Affinity Chromatography (IMAC) Resin charged with Ni^2+^, Econo-Column^®^ Chromatography Columns and Clarity^™^ ECL Western Blotting Substrate were purchased from Bio-Rad Laboratories (Hercules, CA, USA). HiLoad 16/600 Superdex 75 pg gel filtration chromatography column was purchased from GE Healthcare (Chicago, Illinois, USA). Amicon Ultra concentrator with a 10 kDa cut-off filter was obtained from Merck Millipore (Darmstadt, Germany). Immun-Blot PVDF Western Blotting Membrane was purchased from Bio-Rad Laboratories (Hercules, CA, USA). His-Tag (D3I10) XP^®^ Rabbit Monoclonal Antibody (Cat. 12698) and Anti-Rabbit IgG HRP-linked Secondary Antibody (Cat. 7074) were obtained from Cell Signaling Technology (MA, USA). ADP-Glo^™^ Kinase Assay Kit was purchased from Promega Corporation (Madison, WI, USA). Recombinant ASF1a substrate (Cat. PRO-682) for the kinase assay was purchased from ProSpec Technogene Ltd., Ness-Ziona, Israel.

## Methods

### Construction of hTLK1B-pETDUET-1-Lambda Protein Phosphatase plasmid and expression test

Five hundred and forty-nine amino acids of the Human TLK1B (UniProt- Q9UKI8-3) were optimised for the soluble expression in *E. coli* using Genescript’s OptimumGene^™^ gene design system (US Patent 8,326,547: Document Identifier US 20110081708 A1). To aid in the soluble and homogeneously unphosphorylated expression of our gene of interest, i.e. wild-type hTLK1B in this case, we started with the strategic selection of the plasmid vectors harbouring a co-expression capacity of two target genes. The full-length, 1647 bp coding sequence of hTLK1B cDNA was cloned into the multiple cloning site 1 (MCS-1) of the vector pETDUET-1 in-frame with the N-terminal hexahistidine residues using BamHI and EcoRI, thus adding an MGSSHHHHHH affinity purification tag. The full-length, 663 bp coding sequence of bacteriophage lambda protein phosphatase cDNA was subcloned in MCS-2 of the same vector using MfeI and XhoI. The resulting expression plasmid construct was confirmed by restriction digestion analysis (Supplementary Figure S1) and direct sequencing (SciGenom Labs, Kochi, India) (Supplementary data 1) and was used to transform chemically competent *Escherichia coli* Rosetta Gami 2^™^ (DE3) pLysS cells grown in Luria-Bertani (LB) broth supplemented with 35 μg/ml chloramphenicol and 100 μg/ml ampicillin. Cells were cultured at different temperatures and times, 37 °C for 3 h, 30 °C for 5 h, 25 °C for 9 h and 18 °C for 16 h after induction with series of isopropyl-β-D-1-thiogalactopyranoside (IPTG) concentrations, 0.1 mM, 0.3 mM, 0.5 mM and 0.7 mM when the O. D_600_ was between 0.6 and 0.8. Cells were then harvested by centrifugation, and the pellet was stored at −80 °C until further use.

### Purification of recombinant 6xHis-tagged hTLK1B

The bacterial culture was scaled up to 1 litre with supplementary IPTG (0.5 mM) at 25 °C for 9 hours. To purify the full-length, 64.9 kDa, 6xHis-tagged recombinant hTLK1B protein, the harvested cells were resuspended in a lysis buffer containing 50 mM Tris-Cl (pH-8.8), 500 mM NaCl, 1% Triton-X l00, 10% Glycerol (v/v), 0.3 mM TCEP, 20 mM Imidazole (pH-8.8), and Complete^™^ EDTA-Free Protease Inhibitor tablets. The resuspended cells were lysed by ultrasonication using Vibracell™ VCX-130 cell disruptor (Sonics and Materials Inc., Newtown, CT, USA) at an amplitude of 45% (10 s ON/20 s OFF) while cooling on ice for 7 minutes. The cell suspension was centrifuged at 4 °C at 7800 rev/min for 45 minutes to remove the unlysed cells and debris. The supernatant was loaded onto a pre-equilibrated 3 ml Ni-NTA column (Bio-Rad Laboratories, CA, USA) at a flow rate of 1 ml/min. Unbound material was collected for analysis by SDS-PAGE. The column was washed with 30 column volumes of column wash buffer containing 50 mM Tris-Cl (pH-8.8), 500 mM NaCl, 0.3 mM TCEP, and 50 mM Imidazole (pH-8.8). The bound protein was eluted using an elution buffer containing 50 mM Tris-Cl (pH-8.8), 450 mM NaCl, 200 mM imidazole (pH-8.8), 0.3 mM TCEP and 5% glycerol. The elution fractions were collected, pooled together and concentrated using Amicon Ultra concentrator (Millipore) with a 10 kDa cut-off filter. The concentrated protein sample was then further purified and buffer-exchanged into 50 mM Tris-Cl (pH-6.5), 50 mM NaCl and 0.2 mM TCEP by size-exclusion chromatography (SEC) on a HiLoad 16/60 Superdex 75 column (GE Healthcare, Chicago, Illinois, USA). The homogeneity of the protein was determined by Coomassie Blue staining of a 12% SDS-PAGE gel **(**Supplementary Figure, S4).

### Western blot analysis

The protein concentration was quantified by absorbance spectroscopy at 280 nm using calculated extinction coefficient of 58,790 M^−1^ cm^−1^. An equal amount of protein (20 µg) was electrophoresed under the denaturing conditions using 12% SDS gel and 1X Tris-Glycine SDS running buffer with a broad range Precision Plus Protein^™^ Dual Color (Bio-Rad Laboratories, CA, USA) pre-stained protein standard as a molecular size control. The protein sample was electroblotted to an Immun-Blot PVDF Western Blotting membrane (Bio-Rad Laboratories, CA, USA) in an appropriate gel transfer apparatus. The membrane blot was blocked in 5% (w/v) Fat-free milk in Tris-Buffered Saline with Tween-20 (TBST) to reduce non-specific binding. The membrane blot was carefully washed and rinsed with 1X TBST wash buffer and incubated with His-Tag (D3I10) XP^®^ Rabbit Monoclonal Antibody^18^ (Cell Signaling Technology, MA, USA; 1:10000 in blocking buffer) overnight at 4 °C on a rocking platform. After the reaction with an Anti-Rabbit IgG Horseradish Peroxidase-linked Secondary Antibody^19^ (Cell Signaling Technology, MA, USA; 1:5000 in 1 × TBST), the protein was detected using ECL as a luminescent substrate according to the manufacturer’s instructions (Bio-Rad Laboratories, CA, USA).

### Circular Dichroism (CD)

Recombinant 6xHis-tagged hTLK1B protein samples (98% purity by SDS-PAGE) were made in a buffer containing 50 mM Tris-Cl (pH-6.5), 50 mM NaCl and 0.2 mM TCEP. The samples were filtered through 0.2-micron filters (Merck Millipore, Darmstadt, Germany) and were free of particulate matter. Concentration-dependent (1 mg/ml, 0.5 mg/ml and 0.3 mg/ml) Far-UV CD spectra of protein samples were recorded in 0.5 mm quartz cuvette at 25 °C using J-815 circular dichroism (CD) spectropolarimeter (Jasco, Inc., MD, USA) equipped with a Peltier-type temperature control system at a wavelength scan of 190 nm to 250 nm **(**Supplementary Figure, S7**)**. The time constant was kept at 4 s with 1 nm bandwidth and a scan rate of 5 nm/min. A signal-averaged over at least three scans were collected. The recorded spectrum was analysed *in-silico* using K2D3 online software, and the secondary structure of recombinant hTLK1B was determined.

### **ADP-Glo**^**™**^**Kinase Assay**

The ADP-Glo^™^ Kinase Assay (Promega, Madison, WI) was performed to assess the biological activity of the recombinant 6xHis-tagged hTLK1B protein. The kinase assay was carried out in a solid white 96-well plate in a volume of 25 µl solvent containing 2.5 µl of 1 mg/ml hTLK1B kinase, 1 µl of 20 µg/20 µl of ASF1a substrate, 2.5 µl of 100 µM ATP (Promega, Madison, WI) and 19 µl of 1 × Kinase Reaction Buffer containing 40 mM Tris-Cl (pH-7.5), 20 mM MgCl_2_ and 0.1 mg/ml Bovine Serum Albumin (BSA). Suitable negative control wells without the protein kinase, substrate and ATP were also included in the kinase assay. Reactions in the each well were started immediately by adding ATP and kept going for half an hour under 30 °C in EnVision multilabel plate reader (PerkinElmer, Inc., MA, USA) incubator chamber without shaking. After the plate was cooled for 5 min at room temperature, 25 µl of ADP-Glo^™^ reagent was added to each well to terminate the kinase reaction and deplete the unconsumed ATP within 40 mins. In the end, 50 µl of kinase detection reagent was added to each well and incubated for 1 hour to simultaneously convert ADP to ATP and allow the newly synthesised ATP to be measured using a luciferase/luciferin reaction. The luminescent signal generated was measured using an EnVision multilabel plate reader (PerkinElmer, Inc., MA, USA) and is proportional to the ADP concentration produced and is correlated with the kinase activity.

### Statistical analysis

The data presented here are the mean ± standard error (SE) of n ≥ 3 of two independent experiments. Statistical analyses were performed using GraphPad Prism 6 statistical software program (GraphPad Software Inc., CA, USA).

## Results

### Expression of recombinant 6xHis-tagged hTLK1B in *E. coli* and its purification

**Figure 1:**
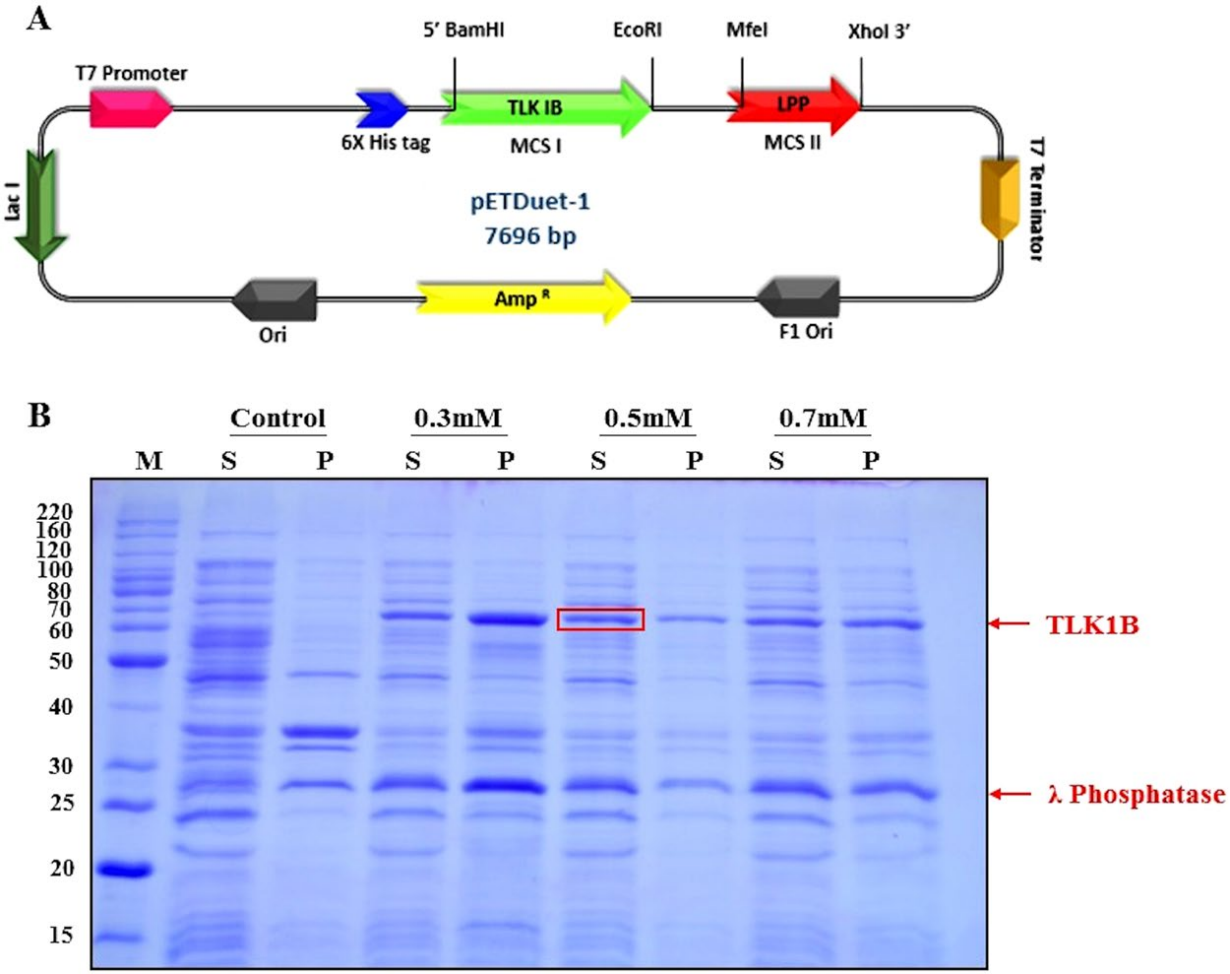
Schematic representation of hTLK1B-pETDUET-1-Lambda Protein Phosphatase expression vector **(A)** and coexpression of recombinant 6xHis-tagged hTLK1B along with bacteriophage lambda protein phosphatase at 25 °C at different IPTG concentrations. **(B)** The full-length hTLK1B gene was fused in-frame with the MGSSHHHHHH affinity purification tag at the N-terminus in the pETDUET-1 vector using BamHI and EcoRI restriction enzymes. The vector contains two multiple cloning sites (MCS), each of which is preceded by a T7 promoter/lac operator and a ribosome binding site (rbs). The vector also carries the pBR322-derived ColE1 replicon, lacI gene and ampicillin resistance gene (AmpR). The full-length, 663 bp coding sequence of bacteriophage lambda protein phosphatase cDNA was subcloned in MCS-2 of the same vector using MfeI and XhoI. The coexpression of the recombinant 6xHis-tagged hTLK1B and bacteriophage lambda protein phosphatase was induced by 0.3 mM, 0.5 mM and 0.7 mM IPTG concentrations at 25-degree Celsius. The control sample was kept uninduced. The abbreviations are as follows: M, molecular weight marker in kDa; S, supernatant; P, pellet.

The hTLK1B-pETDUET-1-Lambda Protein Phosphatase plasmid construct with an MGSSHHHHHH affinity purification tag at the N-terminus shown in Fig. 1A was successfully generated for expression. The vector pETDUET-1 is a bacterial expression vector and was selected for cloning and expression of hTLK1B cDNA in *Escherichia coli* Rosetta Gami 2^™^ (DE3) pLysS cells because of its exceptional properties of coexpression of two target genes^20^. For the first time, *Garrote et al*. *(2014)* expressed and purified TLK2-Kinase domain by co-expressing it along with the bacteriophage λ phosphatase without compromising its yield, solubility and purity^17^. However, homogeneously unphosphorylated soluble expression and purification of full-length, wild-type hTLK1B in a prokaryotic organism such as *E. coli* using such a co-expression strategy have not been reported so far. Until now, the most common method employed for the expression of the active forms of Human Tousled-like kinases (both TLK1 and TLK2) is from the insect cell culture^11^ or with minimal yields in bacteria^12^.

**Figure 2:**
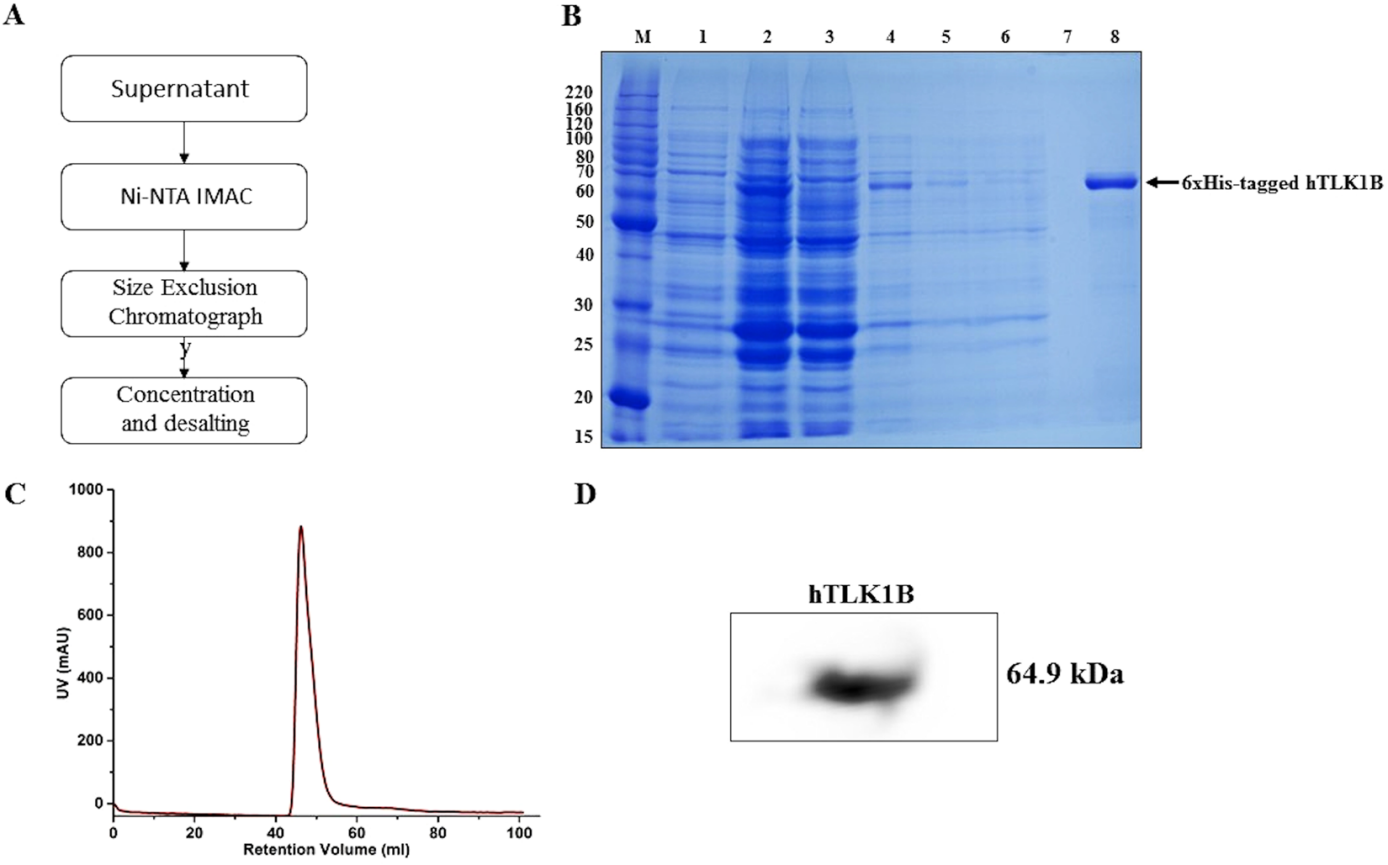
Purification of recombinant 6xHis-tagged hTLK1B from *E. coli*. **(A)** Schematic overview of the purification process using Ni-NTA IMAC and size exclusion chromatography (SEC). **(B)** SDS-PAGE (12% Tris-Glycine gel) analysis of recombinant hTLK1B through different purification steps. M, molecular weight marker in kDa; lane 1, uninduced soluble fraction after cell homogenization (control); lane 2, IPTG induced soluble fraction (total cell lysate); lane 3, unbound protein fraction; lane 4-6, after-binding column wash fractions; lane 8, eluted hTLK1B after 200 mM imidazole wash. Arrow indicates the position of recombinant 6xHis-tagged hTLK1B protein (64.9 kDa). **(C)** Chromatogram of HiLoad 16/60 Superdex 75 size-exclusion column after Ni-NTA IMAC step. **(D)** Immunoblot showing the purified recombinant 6xHis-tagged hTLK1B protein after SEC. His-Tag (D3I10) XP^®^ Rabbit Monoclonal Antibody (Cat. 12698) was used as a primary antibody. The protein was detected by using Anti-Rabbit IgG Horseradish Peroxidase-linked Secondary Antibody (Cat. 7074) and ECL as a luminescent substrate. The blot image has been cropped for clarity and conciseness. The full-length image of the blots (with multiple exposures) have been included in the Supplementary Figure, S5.

In order to check the expression levels of recombinant 6xHis-tagged hTLK1B in *E. coli* by bacteriophage λ phosphatase co-expression approach, several induction conditions including temperature and time (37 °C for 3 h, 30 °C for 5 h, 25 °C for 9 h and 18 °C for 16 h) and different IPTG concentrations (0.1 mM, 0.3 mM, 0.5 mM and 0.7 mM) were tested. As a result, the expression of protein at 25°C for 9 h with 0.5 mM IPTG concentration showed high soluble expression efficiency of approximately 50–60% respectively (as observed in 12% SDS-PAGE) in the supernatant fraction **(**Fig. 1B**)**. The resulting supernatant fraction was further loaded onto a pre-equilibrated 3 ml Ni-NTA column (Bio-Rad Laboratories, CA, USA) at a flow rate of 1 ml/min and the recombinant 6xHis-tagged hTLK1B was purified using Immobilized metal affinity chromatography (IMAC) purification technique. IMAC is a powerful purification technique which relies on the affinity of the clustered histidine residues towards charged transition metal ions such as Ni^+2^ in this case, immobilised on the chelating surface such as a nitriloacetic acid (NTA)^21^. The bound protein was eluted using a concentration gradient of imidazole ranging from 100 mM up to 500 mM^22^ **(**Supplementary Figure, S3**)**. We found that 200 mM concentration of imidazole is sufficient to elute the bound protein competitively **(**Fig. 2B**)**. The eluted fractions were pooled together and further purified by size-exclusion chromatography (SEC) on a HiLoad 16/60 Superdex 75 column **(**Fig. 2C**)**.

Fractions of the protein purified by SEC were collected, concentrated to approximately 6 mg/ml and found to be nearly 98% pure **(**Fig. 2C and Supplementary Figure, S4C**)**. The concentration of protein was estimated by absorbance spectroscopy at 280 nm using calculated extinction coefficient of 58,790 M^−1^ cm^−1^^23^. The protocol yields about 6–12 mg of purified recombinant hTLK1B protein per litre of the bacterial culture. The pH-8.8 of the buffers used for purification were very close to the theoretical isoelectric point (pI) of the protein, i.e. 8.68. Around this pH, the protein becomes less soluble as there is no net charge and it tends to aggregate. Therefore, to prevent the aggregation of the protein and to ensure solubility, the protein was buffer-exchanged into 50 mM Tris-Cl (pH-6.5), 50 mM NaCl and 0.2 mM TCEP. The purified recombinant hTLK1B protein was subjected to mass spectrometric analysis **(**Supplementary Figure, S6**)** to confirm its identity. High-resolution mass spectrometry (LC-MS-MS) is a routinely used technique for the analysis of protein phosphorylation^24^. The selected Coomassie-stained protein band was excised from the SDS gel, destained and the identity of the recombinant hTLK1B protein was confirmed by in-gel trypsin digestion (Proteomics facility, IISc Bangalore, Karnataka, India). Raw data files **(**Supplementary data 2**)** were submitted to MASCOT Peptide Mass Fingerprint server (Matrix Science Inc., MA, USA)^25^, and a non-redundant Swiss-Prot database containing 552,259 human protein sequences was searched using the following parameters: Instrument type was selected using default precursor settings with a specified mass tolerance of 0.5 Da (fragment), Cysteine carbamidomethylation was included as a fixed modification and methionine oxidation was set as the variable modification, and tryptic searches were conducted with one missed cleavage permissible. We have found that our recombinant hTLK1B is completely dephosphorylated due to co-expression of lambda protein phosphatase and is in alignment with the native protein sequence. The purified, recombinant hTLK1B protein was further subjected to immunoblotting using HRP-conjugated secondary antibody and ECL as a detection reagent which again validated the identity of the protein **(**Fig. 2D**)**.

### Secondary structure determination of the purified, recombinant hTLK1B by circular dichroism (CD)

**Figure 3:**
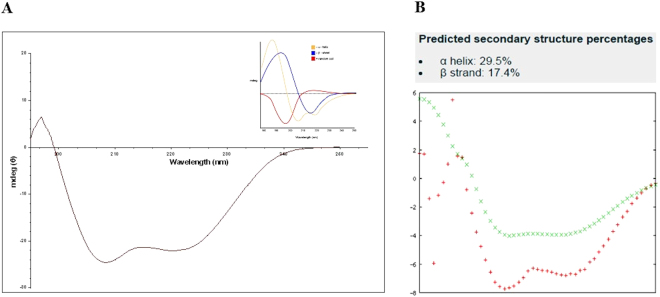
The circular dichroism (CD) spectrum of recombinant 6xHis-tagged hTLK1B protein **(A)**. The protein was made in buffer containing 50 mM Tris-Cl, 50 mM NaCl and 0.2 mM TCEP at pH-6.5 in 0.3 mg/ml concentration. The spectrum was recorded in 0.5 mm quartz cuvette at 25 °C using J-815 circular dichroism (CD) spectropolarimeter (Jasco, Inc., MD, USA). The CD spectrum is obtained from the data sets of three individual replicate experiments and the mean residue ellipticity (θ) was fit using GraphPad Prism software (Version 6.01, La Jolla, CA). The data confirms the presence of alpha helices (29.5%) and beta sheets (17.4%) in the secondary structure of hTLK1B. Inset: Representative protein secondary structures (α-helix: Yellow; β-sheet: Blue; and random coils: Red) with varying conformations. *In-silico* K2D3 analysis of hTLK1B **(B)**.

The secondary structure composition and the folding properties of the purified, recombinant hTLK1B protein were estimated by circular dichroism (CD) **(**Fig. 3**)**. Knowledge of the protein conformation is fundamental for understanding numerous biological processes. With the advent of CD instrumentation in the recent years, circular dichroism (CD) has become increasingly recognised as a valuable structural technique to study proteins in the physiological conditions in which they operate, i.e. in the solution^26^. Since the spectra of proteins are so dependent on their conformation, CD can be used to monitor conformational changes and estimate the structure of unknown proteins such as hTLK1B. The underlying principle of the CD-spectroscopy states that when the chromophores of the amides of the polypeptide backbone of proteins are located in a naturally folded environment, their optical transitions are shifted or split into multiple transitions due to ‘exciton’ interactions. The result is that the different structural elements have characteristic CD spectra^27^. Alpha helix, beta sheets and random coil structures give rise to a characteristic shape and magnitude of CD spectrum^26,27^. Thus, the relative fraction of each secondary structure type that is present in any protein can be determined by analysing its far-UV CD spectrum. Alpha helix shows negative bands at 222 nm and 208 nm and a positive band at 193 nm whereas beta sheet shows a negative band at 218 nm and a positive band at 195 nm. The random coil has a positive band at 212 nm and a negative band around 195 nm^26,27^. The CD spectra of the recombinant hTLK1B protein showed a minimum at 220 nm and 208 nm which is a characteristic of alpha-helix proteins **(**Fig. 3A**)**. Comparatively, a 220 nm dip was observed a little higher which indicates that beta-sheets are also present in the structure **(**Fig. 3A**)**. The total number of alpha helices and beta sheets in the recombinant hTLK1B were computed using PSIPRED protein structure prediction server and predicted to be 19 and 14 respectively^28^ (PSIPRED analysis, Supplementary Figure, S8**)**. The total percentage of alpha helices and beta sheets were also calculated *in-silico* using K2D3 software^29^ and were found to be 29.5% and 17.4% **(**Fig. 3B**)**. We found that 0.3 mg/ml concentration of the protein estimated by absorbance spectroscopy at 280 nm using calculated extinction coefficient of 58,790 M^−1^ cm^−1^ is optimum for analysing the CD data **(**Supplementary Figure S7**)**. K2D3 software data significantly correlates with the experimental CD spectra and hence, confirms the presence of alpha helices and beta sheets in the recombinant hTLK1B structure.

### Assessing the bioactivity of the purified, recombinant hTLK1B protein

**Figure 4:**
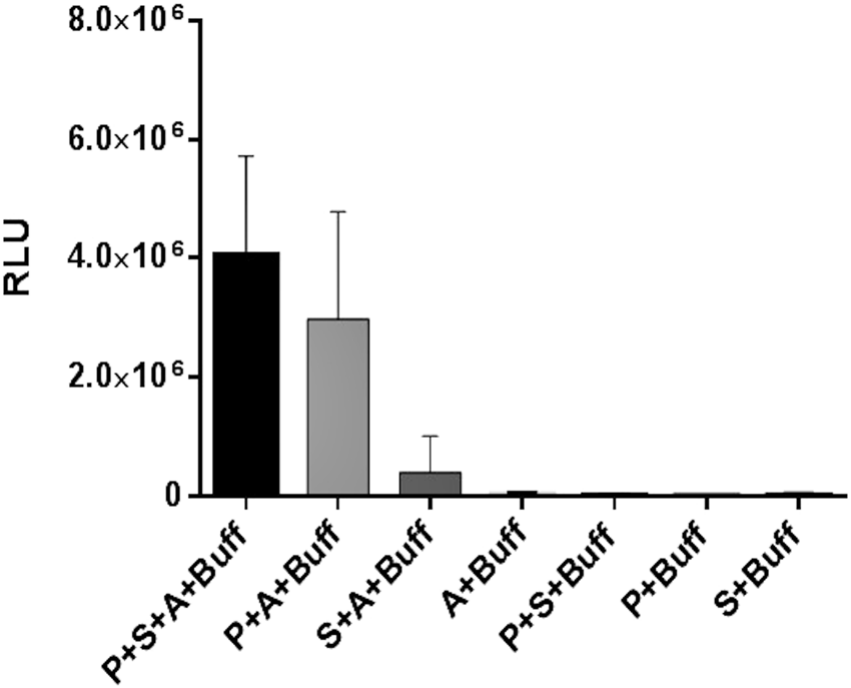
Determination of the bioactivity of recombinant hTLK1B. The ADP-Glo™ kinase assay was performed using 25µl of ADP-Glo reagent and 50 µl of kinase detection reagent at 30 °C in a solid, white 96-well plate as described previously in the Materials and Methods section. Luminescence values represent the mean of three replicates. The abbreviation are as follows: P, recombinant hTLK1B kinase protein; S, ASF1a kinase substrate; A, Adenosine triphosphate; Buff, 1x kinase reaction buffer; RLU, Relative light units.

To determine the biological activity of the purified, recombinant hTLK1B protein, a novel ADP-Glo^™^ Kinase Assay which employed coupled luminescence measurements of ADP formation was performed^30^. The assay was done in two steps upon completion of a kinase reaction: a combined termination of kinase reaction and depletion of remaining ATP in the first step, and conversion of generated ADP to ATP and the newly produced ATP to light output using luciferase/luciferin reaction in the second step. The amount of light generated is proportional to the ADP produced and the activity of the kinase. The kinase reaction was carried out at 30°C for 30 mins, and all 96-well assay plates were read using an EnVision multilabel plate reader (PerkinElmer, Inc., MA, USA). The instrument was set to the 0.5 s integration time. To estimate the amount of ADP produced in the kinase reaction, we created a standard curve that represented the luminescence corresponding to the conversion of ATP to ADP (the “ATP-to-ADP conversion curve”) based on the ATP concentration used in the kinase reaction **(**Supplementary Figure 9A**)**. The conversion curve represents the amounts of ATP and ADP available in the reaction at the specified conversion percentage. The ATP to ADP conversion curve was also used to assess the linearity of the assay and during enzyme titrations to calculate the amount of ADP produced from each amount of enzyme used **(**Supplementary Figure 9B**)**. To plot, analyse the data and calculate all kinase reaction biochemical values, both Microsoft Excel and Prism from GraphPad software (La Jolla, CA) were used. We found that the purified, recombinant hTLK1B is biologically active and showed the luminescence intensity of 4.05 × 10^6^ RLU **(**Fig. 4, Bar 1 from left). However, surprisingly, one of the negative controls without substrate containing only protein kinase, ATP and 1 × kinase reaction buffer also showed comparable luminescence signal of 3.05 × 10^6^ RLU **(**Fig. 4, Bar 2 from left). We reason that this undesirable luminescence signal could be because of the hTLK1B’s propensity to dimerise and autophosphorylate^11^. The specific kinase activity (with respect to ADP production) was calculated at the varying concentrations of hTLK1B protein ranging from 0 µM to 1.5 µM with a k_cat_ value of 0.067 min^−1^ (for autophosphorylation) and 0.13 min^−1^ (for Asf1a substrate phosphorylation). As the enzyme concentrations increased, a subsequent increase in the ADP production and the kinase activity was also observed. About 80% of ADP was produced at the enzyme concentration of 1.5 µM **(**Supplementary Figure, 9B, Blue curve). Similar trend was observed with respect to the hTLK1B autophosphorylation experiment that was performed in the absence of substrate. However, the amount of ADP produced in case of autophosphorylation was significantly lower (~40% ADP production) than that of the kinase activity (in the presence of substrate) **(**Supplementary Figure, 9B, Red curve). These observations exactly corroborate with the data that is represented in Fig. 4 (Bar 1 and Bar 2 from left). A growing number of studies have shown that autophosphorylation is an important regulatory step in the activation of many eukaryotic protein kinases^31^. Human TLKs have been described previously by *Silljé, H. H. W. et. al. (1999)* to dimerise and autophosphorylate^11^. Surprisingly, this key regulatory reaction is still poorly understood in TLKs. In order to examine that the comparable luminescence signal was because of kinase autophosphorylation, in our study, we utilised Thioridazine (THD), as a positive control and a known inhibitor for TLK autophosphorylation^4^ **(**Supplementary Figure, 9C**)**. THD belongs to the class of phenothiazine antipsychotics and it does not resemble ATP. It works precisely at the low micro-molar (10 µM) concentrations to inhibit TLK autophosphorylation, however, the mode of binding of THD to TLKs is yet unclear. This was validated using IP/autokinase/autoradiography assays respectively by Ronald, S. *et al*.^4^. Alternatively, in our work, we performed ADP-Glo™ kinase assay to investigate hTLK1B autophosphorylation. Precisely, we also observed that, at 10 µM concentration of THD, the hTLK1B autophosphorylation was drastically inhibited **(**Supplementary Figure, 9A**)**. Our results correctly match with the information described in the published report on TLK1/1B autophosphorylation. Based on these observations, we confirm that the comparable luminescence was due to hTLK1B autophosphorylation. It is not surprising to observe the autophosphorylation mechanism in TLKs, since almost all protein kinases catalyse autophosphorylation reaction, and their conformational changes are frequently regulated by phosphorylation and other regulatory ligands^32^. According to the literature, TLK’s (TLK1/1B and TLK2) work as dimers^11^. Furthermore, previous work using ^32^P incorporation into TLK proteins *in-vitro* revealed TLK autophosphorylation, a common mode of regulation amongst kinases^11,33^. Most likely, the autophosphorylation is hypothesised to be an intra- or intermolecular autophosphorylation^34^. However, the detailed kinetic analysis of autophosphorylation, the number and type of sites/residues which are autophosphorylated and whether they precede the activation of the kinase activity still remains unknown and is an open question to address. Data from the Guillermo Montoya (CNIO) and Anja Groth (BRIC) laboratories using different expression methods identified four putative TLK2 autophosphorylation sites: TLK2-**S**569, TLK2-**S**635, TLK2-**S**686 and TLK2-**S**617. Further evidence on TLK2 autophosphorylation comes from the thesis work of Helena Gonzalez Buron (University of Barcelona, Spain) who confirmed that the residue **S**635, located in the TLK2 activation loop, is a crucial TLK2 autophosphorylation site and could be required for TLK2 activity. However, further cellular assays are necessary to validate its functional relevance^35^. Since the kinase domain is highly conserved between TLK1/1B and TLK2, we predict a corresponding residue in TLK1/1B may be involved in its activation and autophosphorylation. Hence, it is possible the kinase is only hydrolysing ATP. Nevertheless, further experimentation is required to assess its involvement in the process. Based on the experimental data **(**Supplementary Figure 9A–C**)** and observations, we understand that our recombinantly expressed hTLK1B is very moderately active (even at high concentrations tested) at the conditions in which the experiment was conducted. Since the majority of the estimated 20,000 enzymes in the human cells have not been yet characterised thoroughly, it is indeed possible that a few will emerge that do have k_cat_ values somewhat below 1 min^−1^. We also speculate that our recombinantly expressed hTLK1B kinase behaves similarly to the unphosphorylated form of mitogen-activated protein kinase p38α^36^, KaiC^37^ and CaMKII^38^ kinase reported in the literature having a low catalytic efficiency (k_cat_ of 1.1 ± 0.03 min^−1^, 10^–3–10–4^ min^−1^ and 8 × 10^−6^ min^−1^) in contrast to the human Src kinase having a higher catalytic ability (k_cat_ of 500 min^−1^)^39^. We, for the first time, report a method for bacterial expression and purification of the full-length, wild-type hTLK1B by co-expressing it along with bacteriophage lambda protein phosphatase. As the hTLK1B is a ‘less-explored’ kinase with very little information available on its substrates and the interaction partners, an in-depth study is yet required for a detailed understanding of the mechanism and regulation of this enzyme which remains a point of discussion of the.

## Discussion

In this study, for the first time, we have described in detail the soluble prokaryotic and homogeneously unphosphorylated expression of full-length, wild-type recombinant 6x His-tagged hTLK1B using a bacteriophage λ phosphatase co-expression strategy and its simple purification by conventional immobilised metal affinity chromatography technique. The expression and purification procedures employed are reproducible, and the resulting protein is stable. When we initiated a project to establish a bacterial expression system for hTLK1B, we found that kinases that are expressed in high yields in bacteria (~1000 mg/L of TB) often tend to form insoluble precipitates. Prokaryotic cells are usually the preferred hosts for the expression of heterologous proteins due to relatively low cost and speed of production along with the high yields. However, there are frequent problems with poor solubility, incorrect folding or aggregation of the expressed proteins^13^. Many kinases suffer from such problems when expressed in prokaryotic hosts and also, can undergo auto-phosphorylation producing heterogeneously phosphorylated species. Several kinases have also been successfully expressed using baculoviral expression vector systems (BEVS)^40^, especially when high quality of correctly folded protein is required, for example, for crystallisation studies. However, undesired heterogeneously phosphorylated kinases are frequently observed when expressed using BEVS due to either phosphorylation by host kinases or autophosphorylation. To address these shortcomings, we found that it was necessary to coexpress a kinase of interest, hTLK1B in this case, along with a bacteriophage lambda protein phosphatase. We chose bacteriophage lambda protein phosphatase because it is highly specific and dephosphorylates not only phospho-Ser and phospho-Thr but also phospho-Tyr in the protein^17,41–44^. Significantly, in our expression procedure, approximately 50–60% of the kinase protein is found in the soluble fraction, and it yields about 6–12 mg of the homogeneously unphosphorylated biologically active hTLK1B protein per litre of the bacterial culture. The protein concentrates readily and can be successfully used for a multitude of biochemical and biophysical studies. The bacterially expressed kinase protein was characterised and was found to be unphosphorylated in alignment with the native protein sequence. However, it can also be autophosphorylated upon incubation with ATP and Mg2^+^ ^45^. Contrastingly, Human TLKs does not contain an RD motif in subdomain VI suggesting that phosphorylation of a threonine in the activation loop may not be required for their activation^46^. The CD analysis demonstrated the presence of 29.5% alpha helices and 17.4% beta sheets in the secondary structure of the protein and validated that the protein is in its properly folded conformation. The *E. coli*-expressed hTLK1B reported to date has always been dispersedly phosphorylated at several Ser/Thr residues^12^. Such interspersed phosphorylation events had made the bacterially expressed protein inappropriate for many kinds of studies, particularly those involving interactions of hTLK1B with regulatory molecules, protein-drugs interactions and enzyme kinetics. Our unphosphorylated recombinant hTLK1B overcomes this current limitation and will aid in understanding the catalytic mechanisms regarding kinetic steps, transition-state stabilisation, substrate specificity, basis of regulation and mechanisms of novel hTLK1B inhibitors. Moreover, it will also help us to compare the effects of phosphorylation events on the activity of unphosphorylated hTLK1B with that of its phosphorylated counterparts. The problem of producing inappropriately activated protein kinases in bacterial expression systems is not unique to hTLK1B. The catalytic subunit of cAMP-dependent protein kinase^47^ and mitogen-activated protein kinases^48^ have been reported to be autophosphorylated in bacterial cells similar to hTLK1B. Since bacteriophage λ phosphatase dephosphorylates not only phospho-Ser and phospho-Thr, but also phospho-Tyr in proteins^44^, bacteriophage λ phosphatase-coexpression system reported here can be applied to other systems, including both serine/threonine kinases and tyrosine kinases, where unphosphorylated forms of bacterially expressed protein have not been obtained. The coexpression system reported here should be a useful contribution in our attempts to analyse the function and determine the three-dimensional structure of unphosphorylated forms of the kinase proteins.

## Electronic supplementary material

Supplementary Information
