# Supplementary material for: High yield bacterial expression, purification and characterisation of bioactive Human Tousled-like Kinase 1B involved in cancer
Source: Sci Rep. 2018 Mar 19;8:4796. doi: 10.1038/s41598-018-22744-5 (PMC5859067; doi:10.1038/s41598-018-22744-5)
Supplement: Supplementary file 1 — Supplementary Information [file 41598_2018_22744_MOESM1_ESM.docx]

**[SREP-17-44726A](http://mts-srep.nature.com/cgi-bin/main.plex?form_type=view_ms&j_id=110&ms_id=186774&ms_rev_no=1&ms_id_key=ftd7MUMjqH2bQrH2zkY0ryBQg)**

**Supplemental Information**

**High yield bacterial expression, purification and characterisation of bioactive Human Tousled-like Kinase 1B involved in cancer**

Siddhant Bhoir^1^, Althaf Shaik^2^, Vijay Thiruvenkatam^1, 3 *^, Sivapriya Kirubakaran^1, 2, *^

^1^Department of Biological Engineering, Indian Institute of Technology Gandhinagar, Simkheda, Palaj, Gandhinagar-382355, Gujarat, India

^2^Department of Chemistry, Indian Institute of Technology Gandhinagar, Simkheda, Palaj, Gandhinagar-382355, Gujarat, India

^3^Department of Physics, Indian Institute of Technology Gandhinagar, Simkheda, Palaj, Gandhinagar-382355, Gujarat, India

***Corresponding Authors:** Dr Sivapriya Kirubakaran, Department of Biological Engineering and Chemistry, Indian Institute of Technology Gandhinagar, Simkheda, Palaj, Gandhinagar-382355, Gujarat, India

Dr. Vijay Thiruvenkatam, Department of Physics and Biological Engineering, Indian Institute of Technology Gandhinagar, Simkheda, Palaj, Gandhinagar-382355, Gujarat, India

**Email:** [priyak@iitgn.ac.in](mailto:priyak@iitgn.ac.in) and [vijay@iitgn.ac.in](mailto:vijay@iitgn.ac.in)

**Fig. S1: Restriction digestion analysis of human TLK1B gene cloned in pETDUET-1 vector**

**
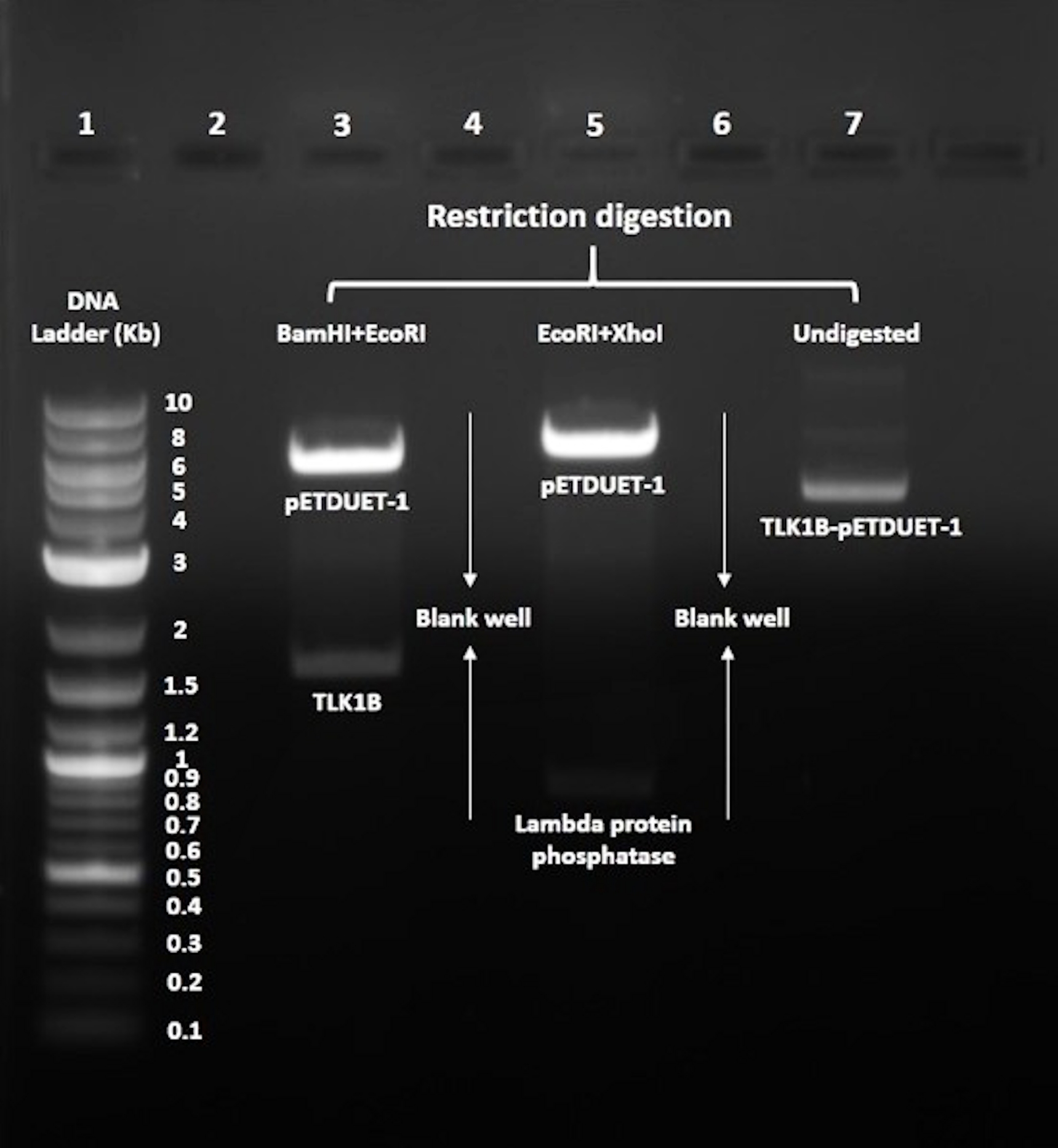
**

**Reaction cocktail 1:**

BamHI-HF- 1 µl

EcoRI-HF- 1 µl

Cutsmart buffer- 2 µl

Plasmid DNA- 20 µl

Nuclease-free water- 6 µl

The total reaction volume was 30 µl

**Reaction cocktail 2:**

EcoRI-HF- 1 µl

XhoI- 1 µl

Cutsmart buffer- 2 µl

Plasmid DNA- 20 µl

Nuclease-free water- 6 µl

The total reaction volume was 30 µl

**Supplementary figure, S1** represents the double-digestion patterns for the restriction endonucleases BamHI-EcoRI (lane 3) and EcoRI-XhoI (lane 5) and the undigested (lane 7) plasmid constructs on an agarose gel. The BamHI-EcoRI digested the plasmid giving two bands, one at 6Kb (digested vector) and the other at 1.6Kb (TLK1B gene). The EcoRI-XhoI gave a banding pattern of 0.6 Kb and 6.4kb respectively. Lane 1, 10kb DNA ladder. Lanes 2, 4 and 6, were intentionally left blank.

**Data 1: Sequencing analysis of human TLK1B gene cloned in pETDUET-1 vector**

**For MCS-1**

**
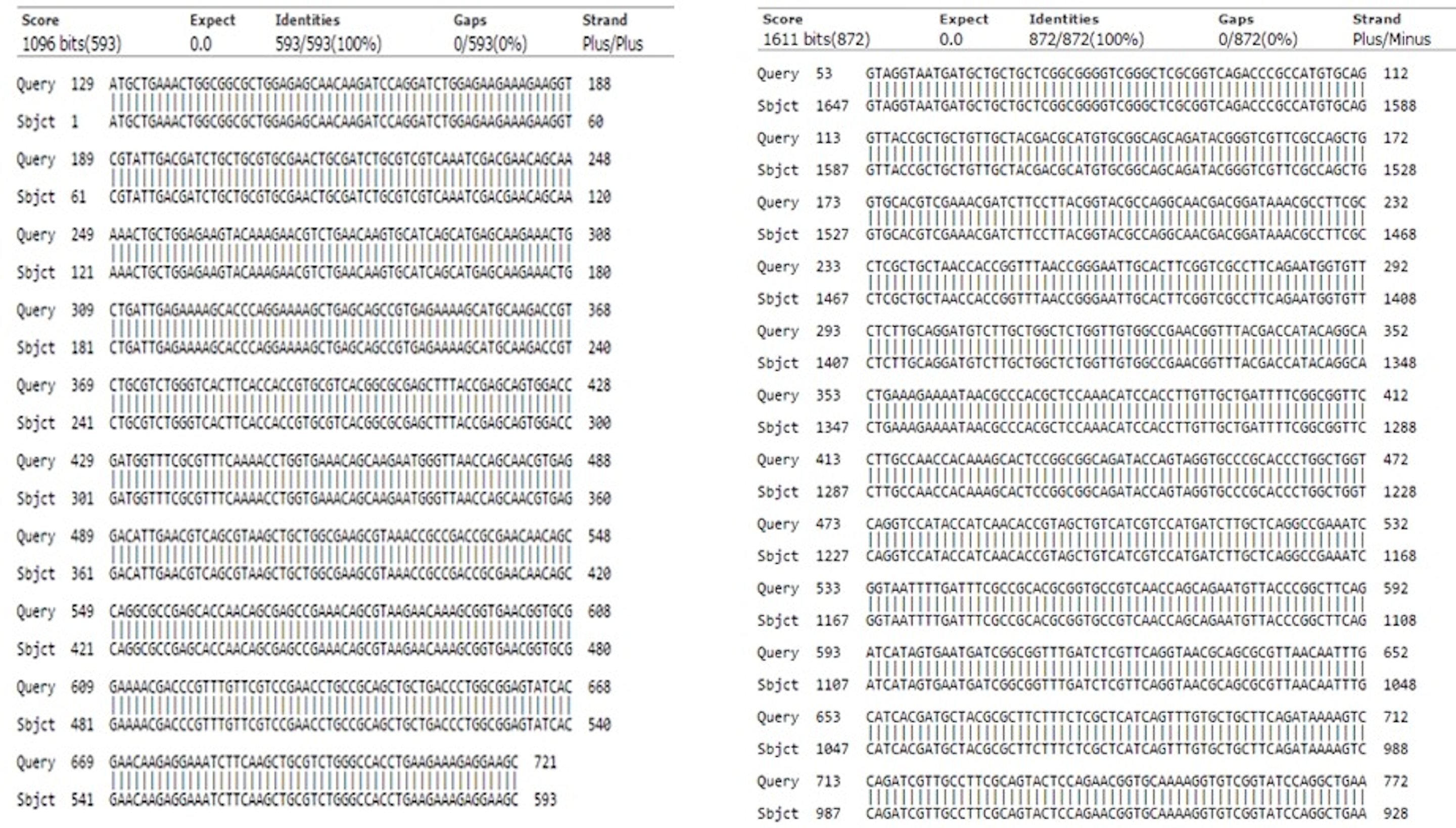
**

**For MCS-2**

**
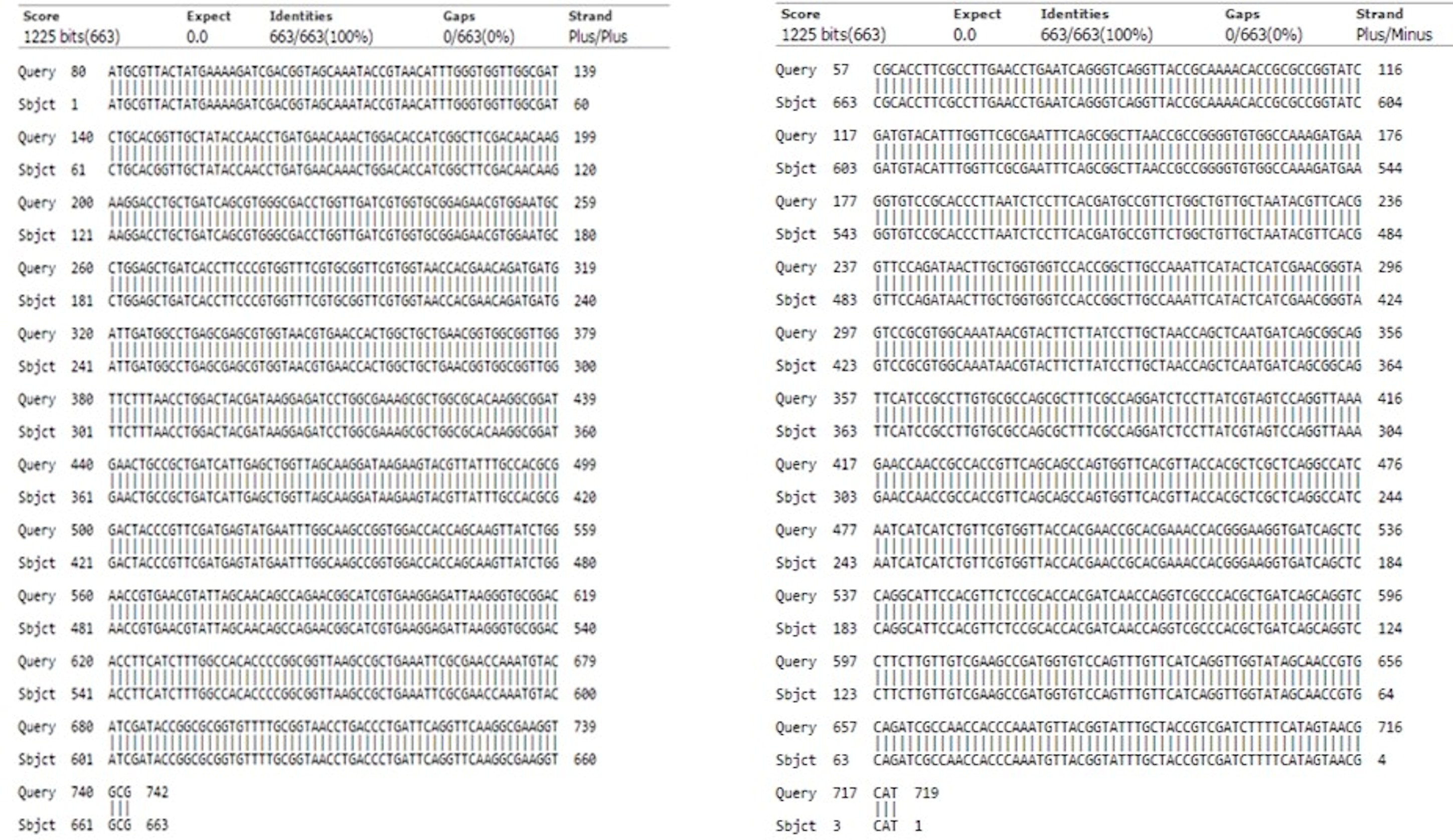
**


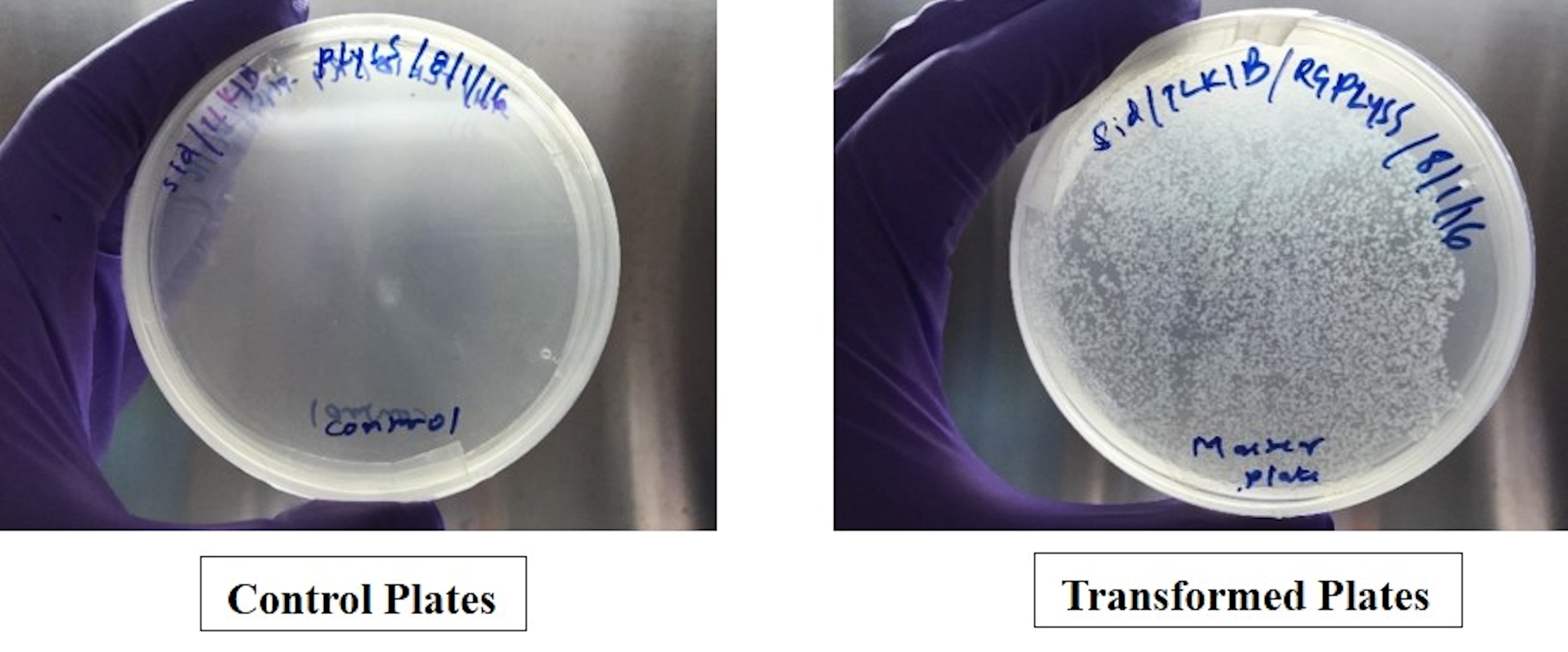
**Fig. S2: Transformation of Human TLK1B in Rosetta gami pLysS 2 *E.Coli* strain**

**Supplementary figure, S2** illustrates the transformation of hTLK1B-pETDUET-1-Lambda Protein Phosphatase vector construct in Rosetta gami pLysS 2 *E.Coli* strain. Control plates were kept untransformed and served as a negative control without any growth. Transformed plates showed growth of the transformed bacterial colonies bearing hTLK1B-pETDUET-1-Lambda Protein Phosphatase construct having resistance to ampicillin.

**Fig. S3: Imidazole gradient hTLK1B purification using Ni-NTA IMAC**


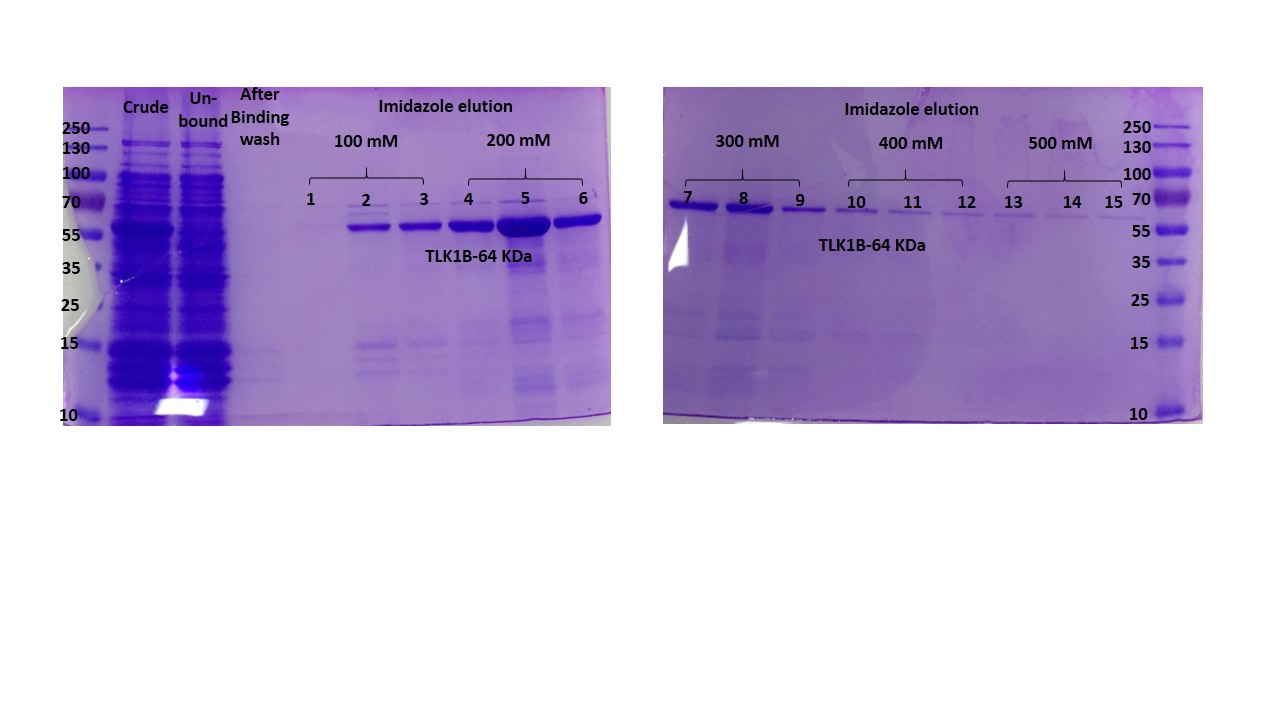

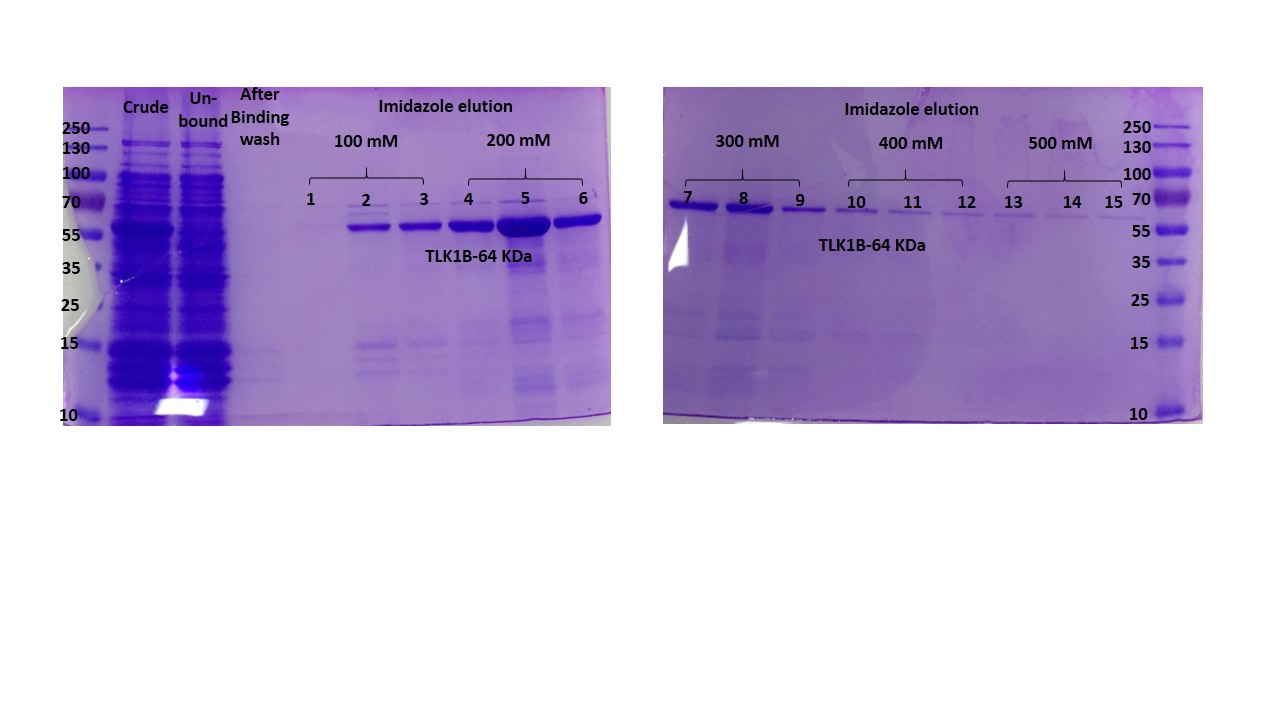


**Supplementary figure, S3** describes the imidazole gradient purification. Lane 1, Molecular weight marker in kDa; Lane 2, crude total cell extract before purification; Lane 3, unbound protein fraction; Lane 4, after binding wash fraction; Lanes 5-7, 100mM imidazole wash fraction; Lanes 8-10, 200mM imidazole wash fraction; Lanes 11-13, 300mM imidazole wash fraction; Lanes 14-16, 400mM wash fraction; Lanes 17-19, 500mM imidazole wash fraction; Lane 20, Molecular weight marker in kDa

**Fig. S4: Size-exclusion chromatography (SEC)-Concentration and Desalting**

A

B

C

**
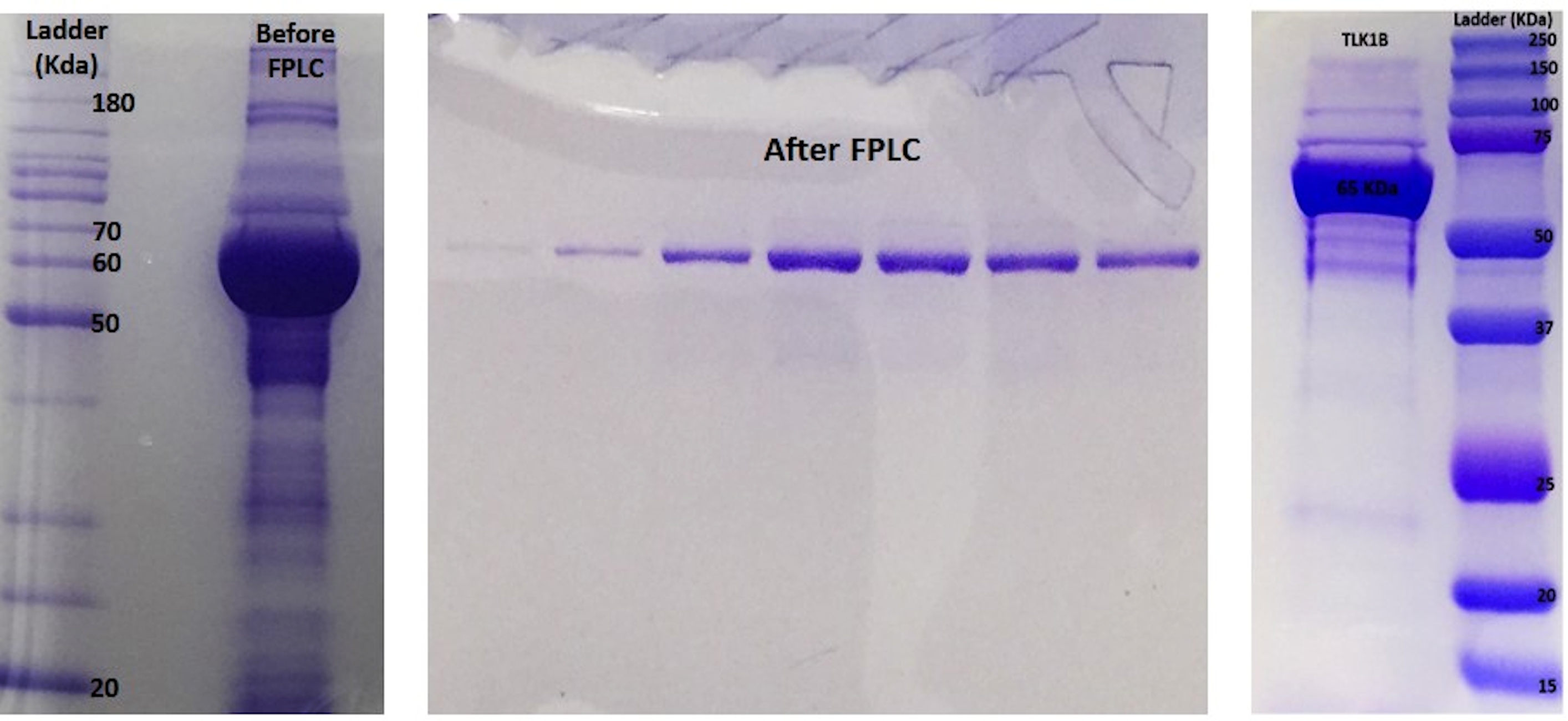
**

**Supplementary figure, S4** shows the Coomassie Brilliant Blue staining of 12% SDS-PAGE after SEC. Gel A, protein sample before purification; Gel B, protein sample after SEC in individual lanes; Gel C, purified and concentrated protein sample (purified fractions pooled together). The elution fractions were collected, pooled together and concentrated using Amicon Ultra concentrator (Millipore) with a 10 kDa cut-off filter. The protein concentration was quantified by absorbance spectroscopy at 280nm using calculated extinction coefficient of 58,790 M^-1^ cm^-1^.

**
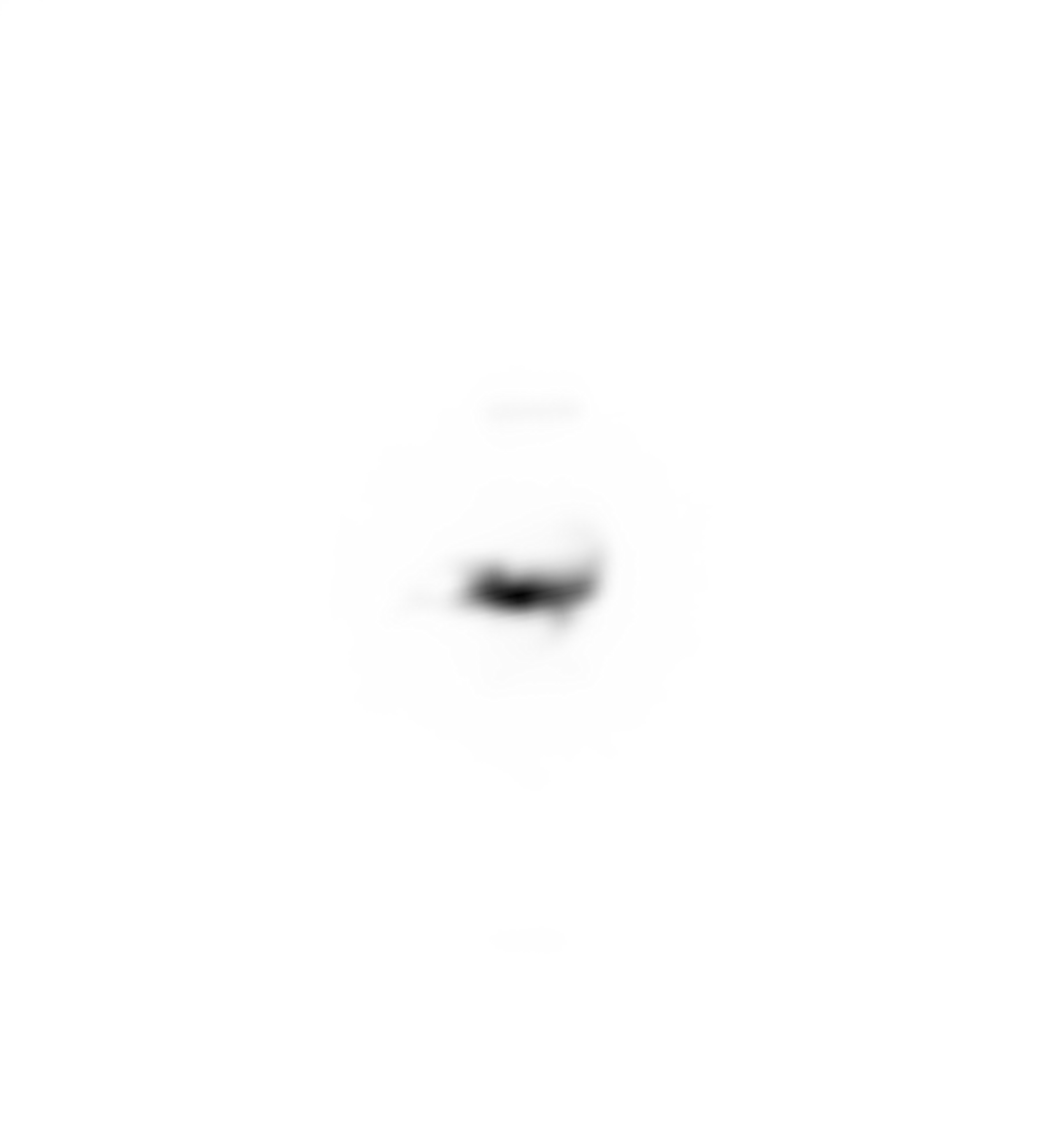
Fig. S5: Immunoblotting of hTLK1B (Multiple Exposures)**

**
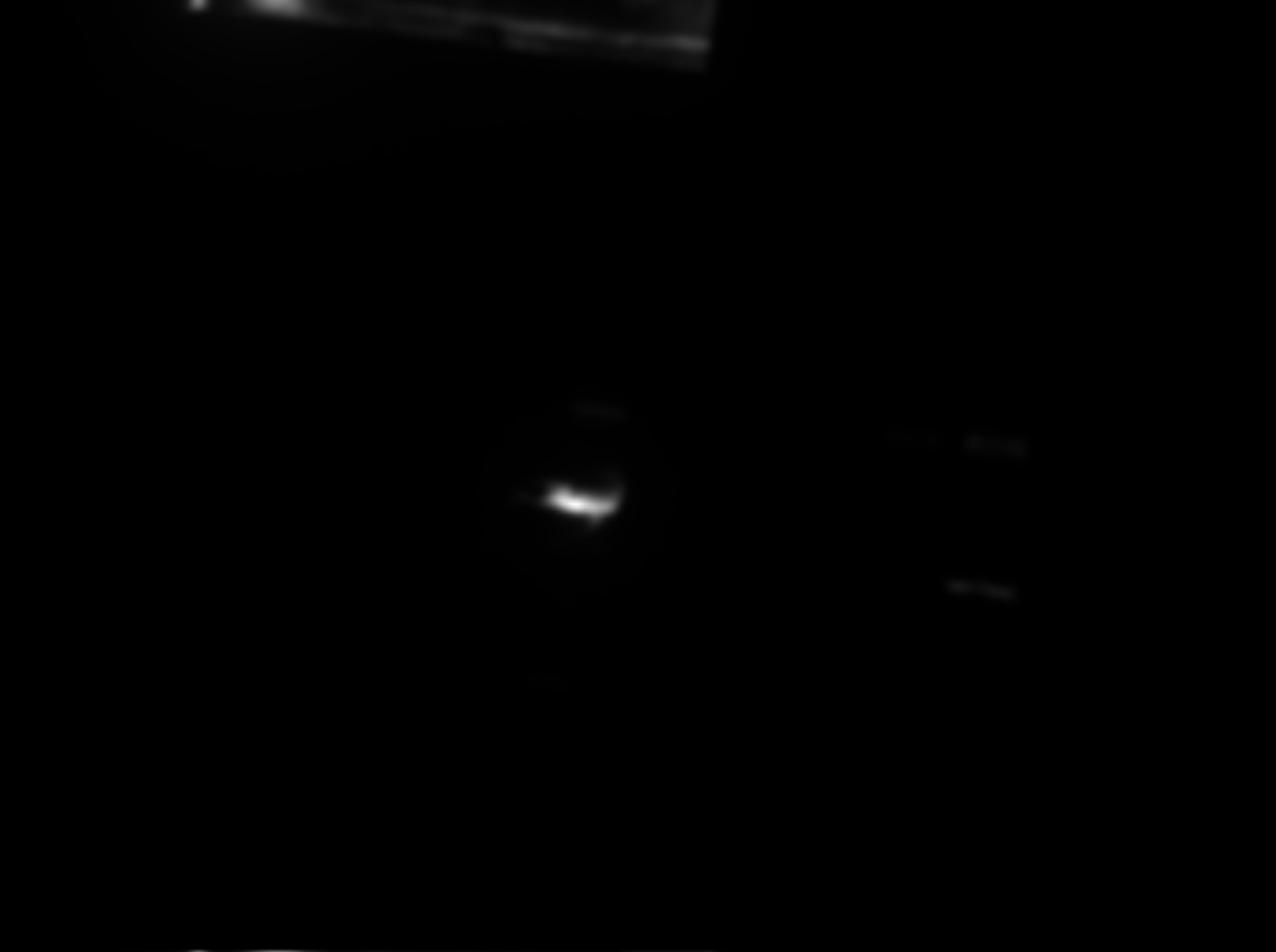

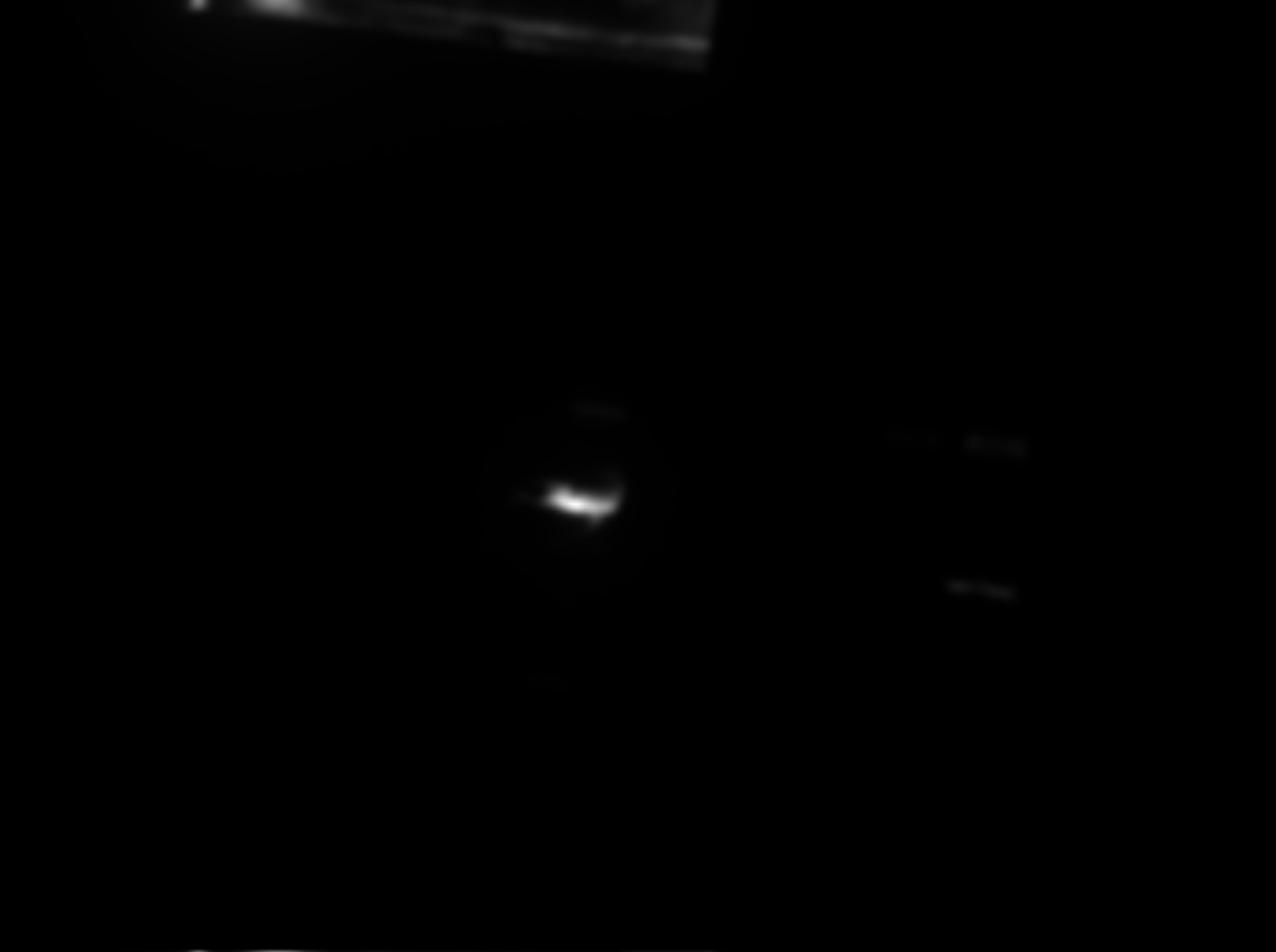
**

**Fig. S6: Mass spectrometry analysis (LC-MS-MS)**


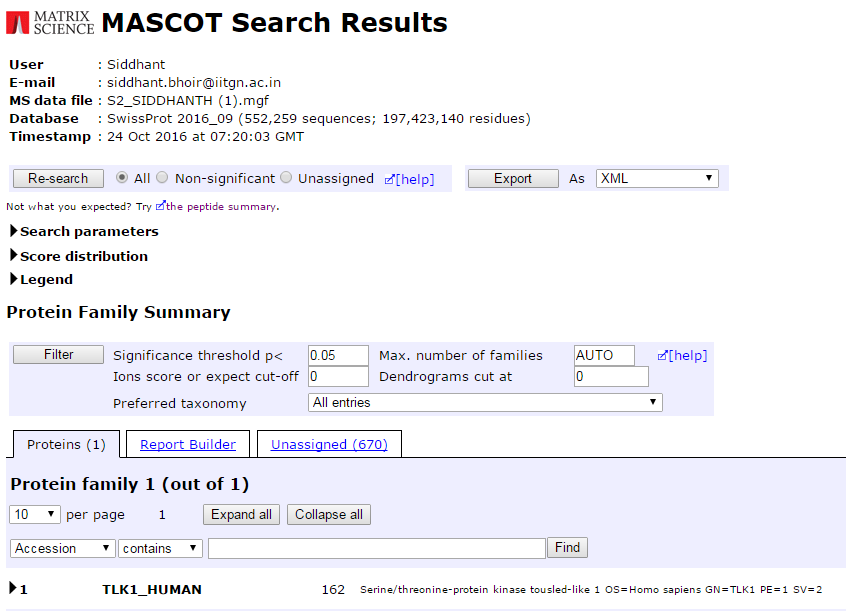


**Supplementary figure, S6** illustrates the mass spectrometry analysis using a MASCOT Peptide Mass Fingerprint Server (Matrix Science Inc., MA, USA) (Ref. 25). The raw data data set (.mgf) of the peptide mass values was submitted to MASCOT Peptide Mass Fingerprint Server (Matrix Science Inc., MA, USA), and a non-redundant Swiss-Prot database containing human protein sequences (552,259) was searched using the following parameters: Instrument type was selected using default precursor settings with a specified mass tolerance of 0.5 Da (fragment), Cysteine carbamidomethylation was included as a fixed modification and methionine oxidation was set as the variable modification, and tryptic searches were conducted with one missed cleavage permissible.


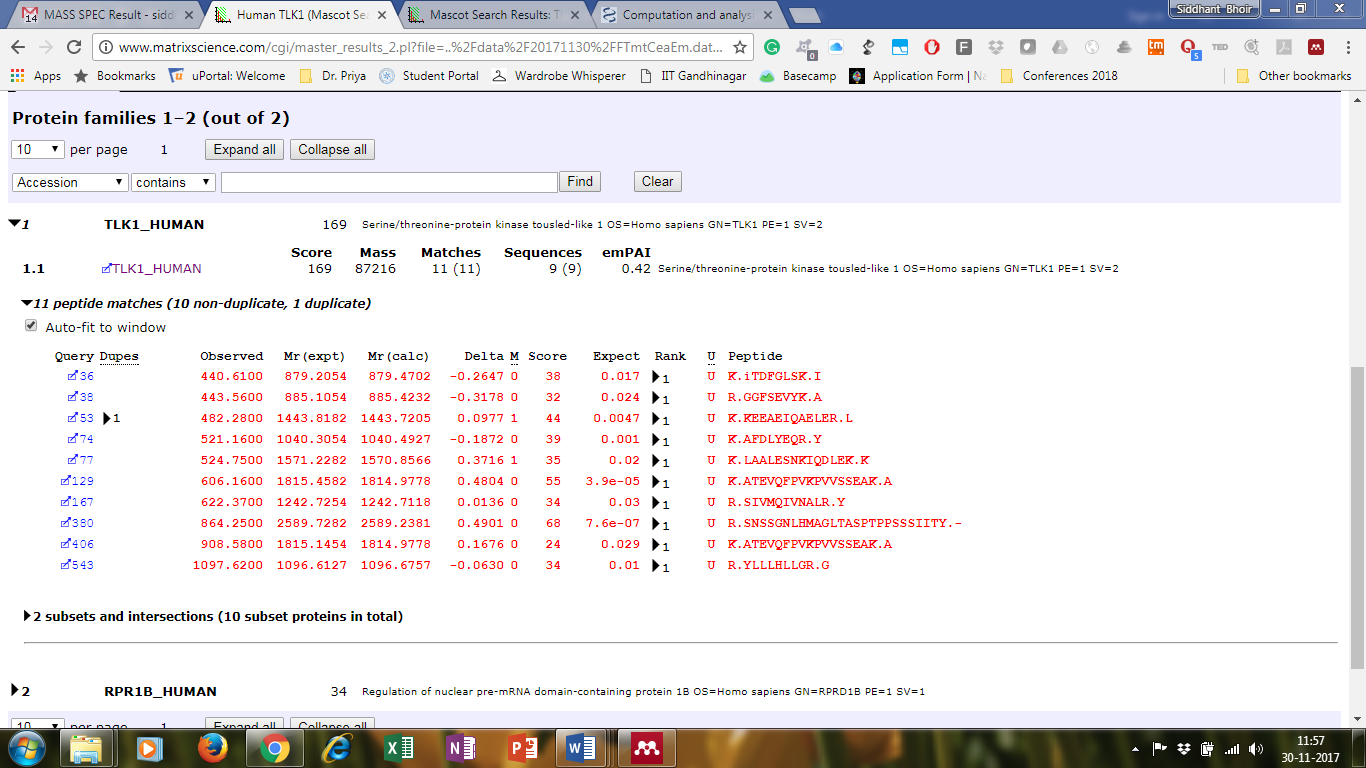


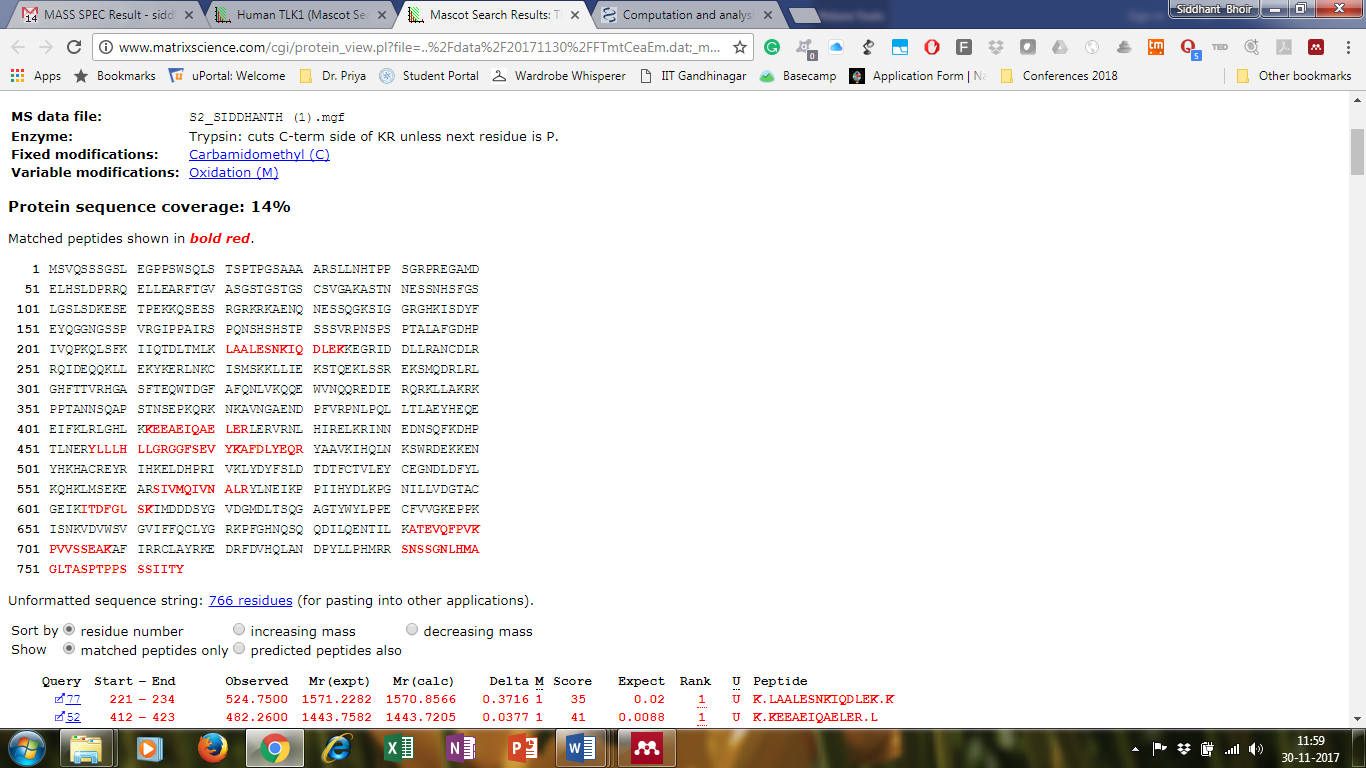


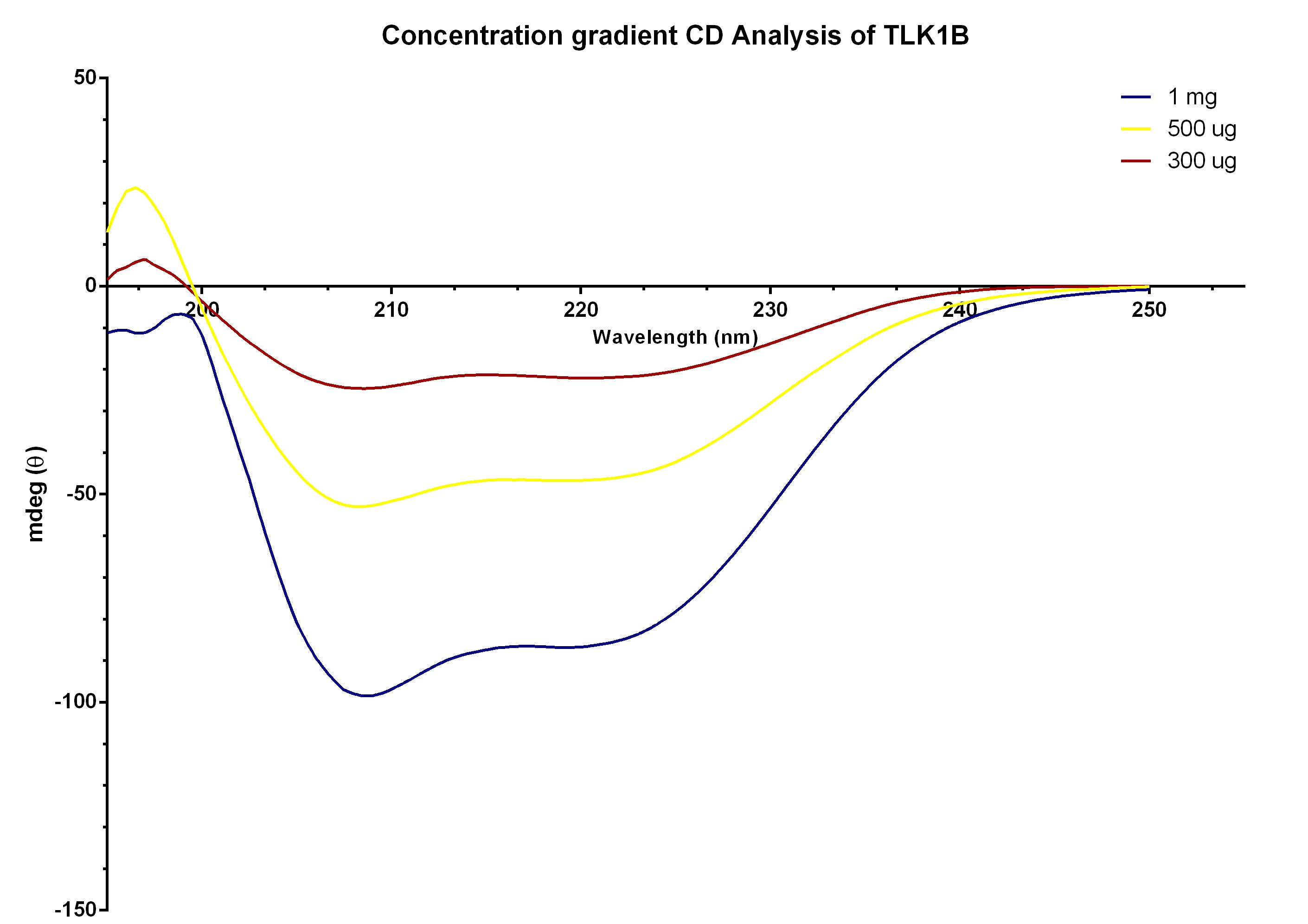
**Fig. S7: CD spectroscopy of recombinant human TLK1B**


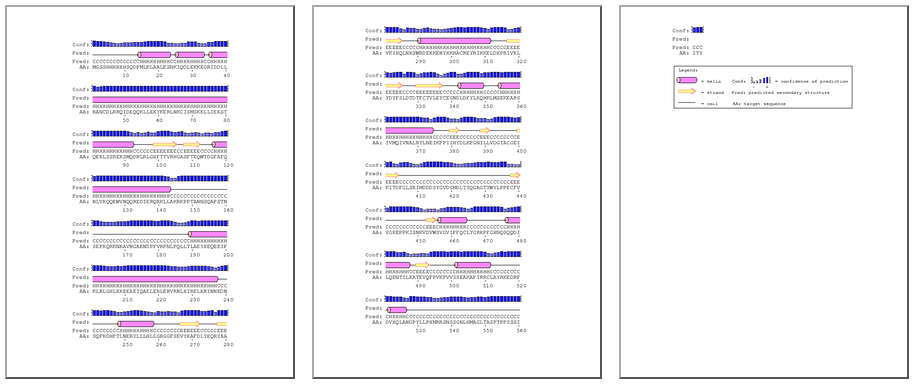
**Fig. S8: PSIPRED analysis of recombinant human TLK1B**


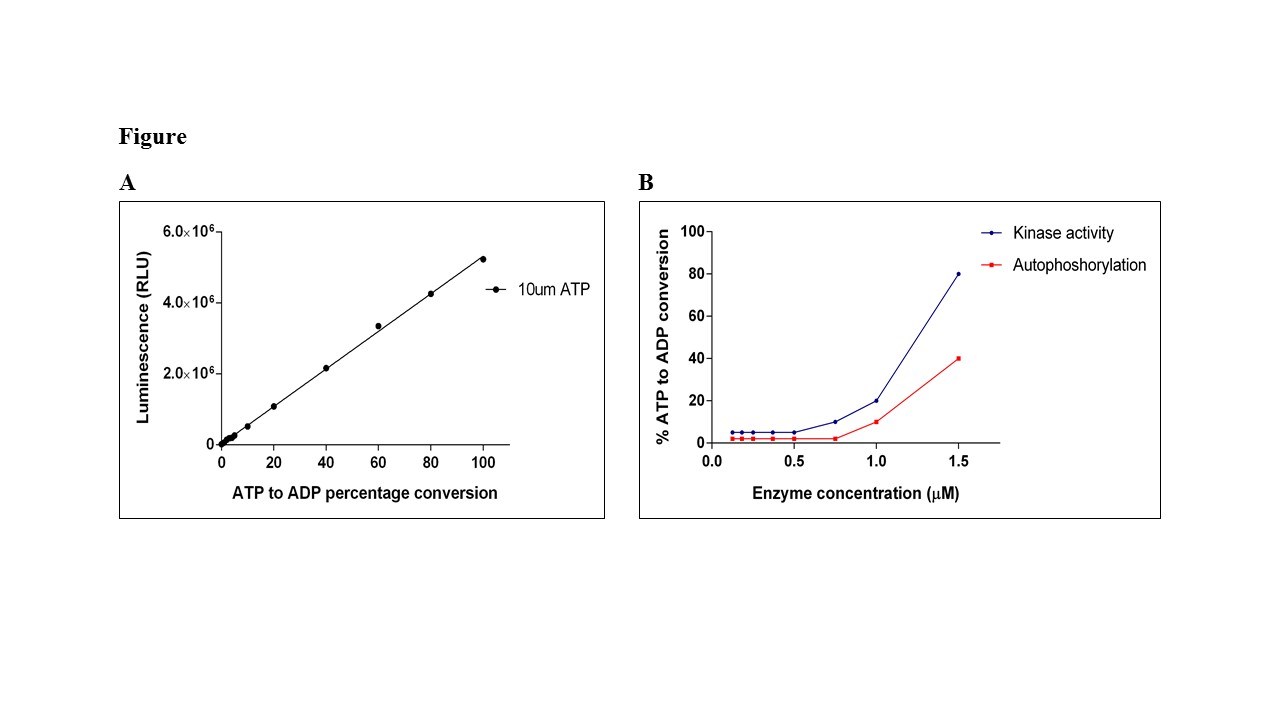
**Fig. S9: ADP-Glo Kinase Assay**

The ATP to ADP standard curve for 10µM ATP **(A).** The ATP-to-ADP conversion curve is created by combining the appropriate volumes of ATP and ADP stock solutions (at the desired ATP+ADP concentrations) in 25µl of 1X reaction buffer in a solid white 96-well plate. ADP-Glo™ Kinase assay was performed using 25µl of ADP-Glo™ reagent and 50µl of Kinase Detection Reagent at room temperature. Luminescence was recorded using an EnVision multilabel plate reader (PerkinElmer, Inc., MA, USA). There is a linear relationship between the luminescent signal and the amount of ADP produced. Assessment of kinase activity and autophosphorylation at different enzyme concentrations **(B).** The kinase assay was carried out in a solid white 96-well plate in a volume of 25 µl of 1X reaction buffer containing varying concentrations of hTLK1B kinase (0µM to 1.5µM), 1 µl of 20 µg/20 µl (1.5µM) of ASF1a substrate, and 2.5 µl of 100 µM ATP. ASF1a substrate was not included in the autophosphorylation reactions. 25µl of ADP-Glo™ reagent and 50µl of Kinase Detection Reagent were added at room temperature and luminescence was recorded after 40 mins. Curve fitting was performed using GraphPad Prism^®^ software


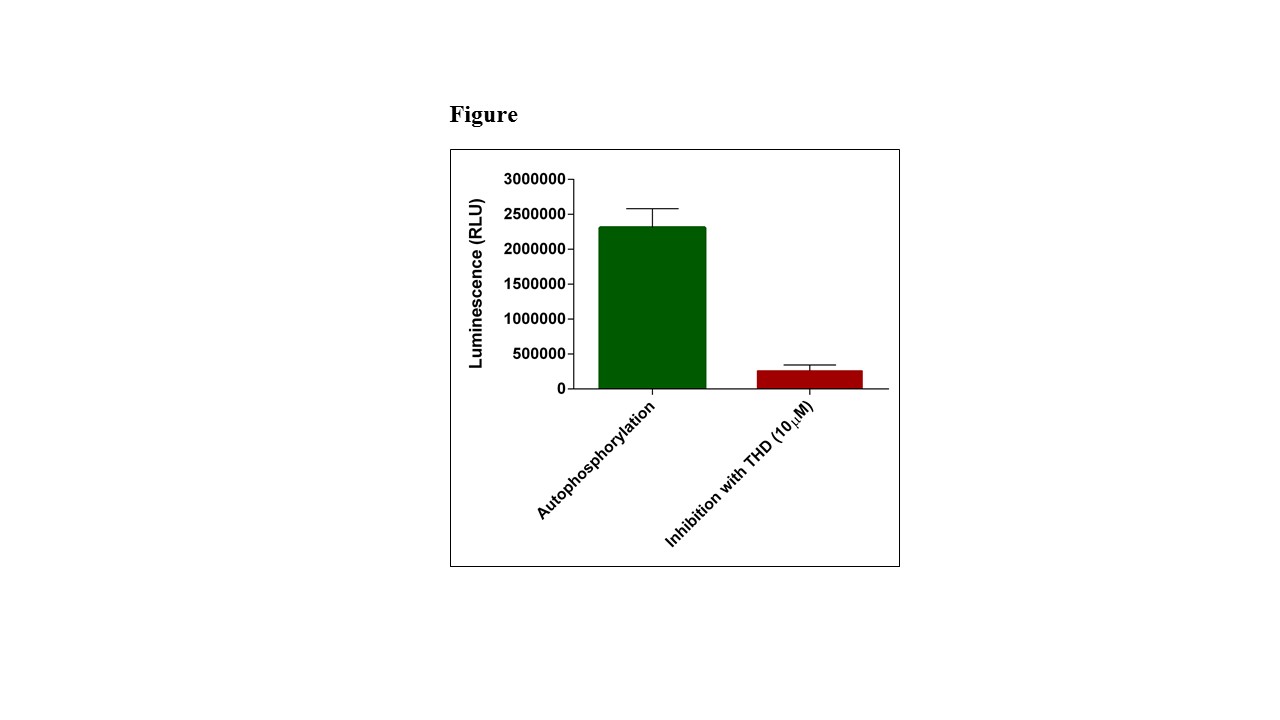


**C**

Inhibition of TLK autophosphorylation by THD. The 25µl kinase autophosphorylation reactions were performed in a solid white flat-bottom 96-well plate in 1X Kinase reaction buffer supplemented with 20 mM MgCl_2_, 0.1 mg/ml Bovine Serum Albumin (BSA), 10µM THD and 10µM ATP at room temperature for 1 hour. Luminescence was detected using 25µl ADP-Glo™ reagent and 50µl Kinase Detection Reagent as described previously. The hTLK1B concentration was kept constant to 1.5µM. The THD inhibitor was not added in the control reaction. GraphPad Prism^®^ software was used to analyse the data.

**Raw gel images**

**
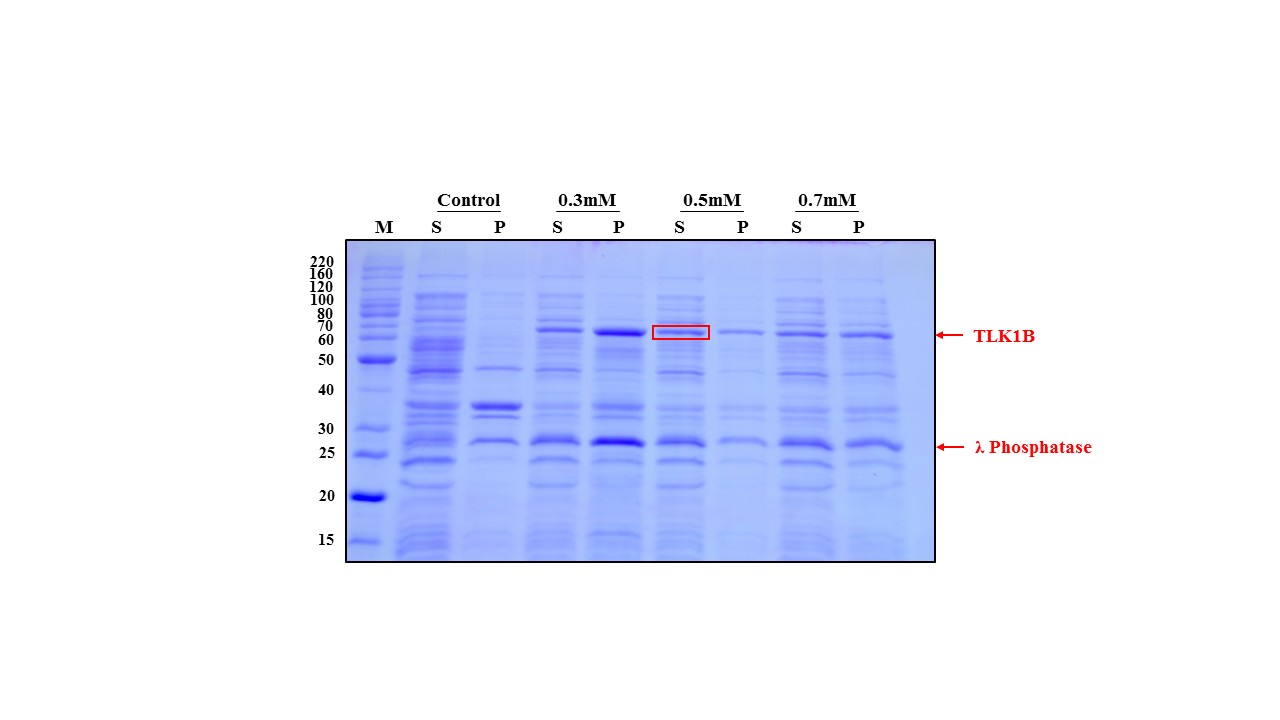
hTLK1B expression and purification**

**
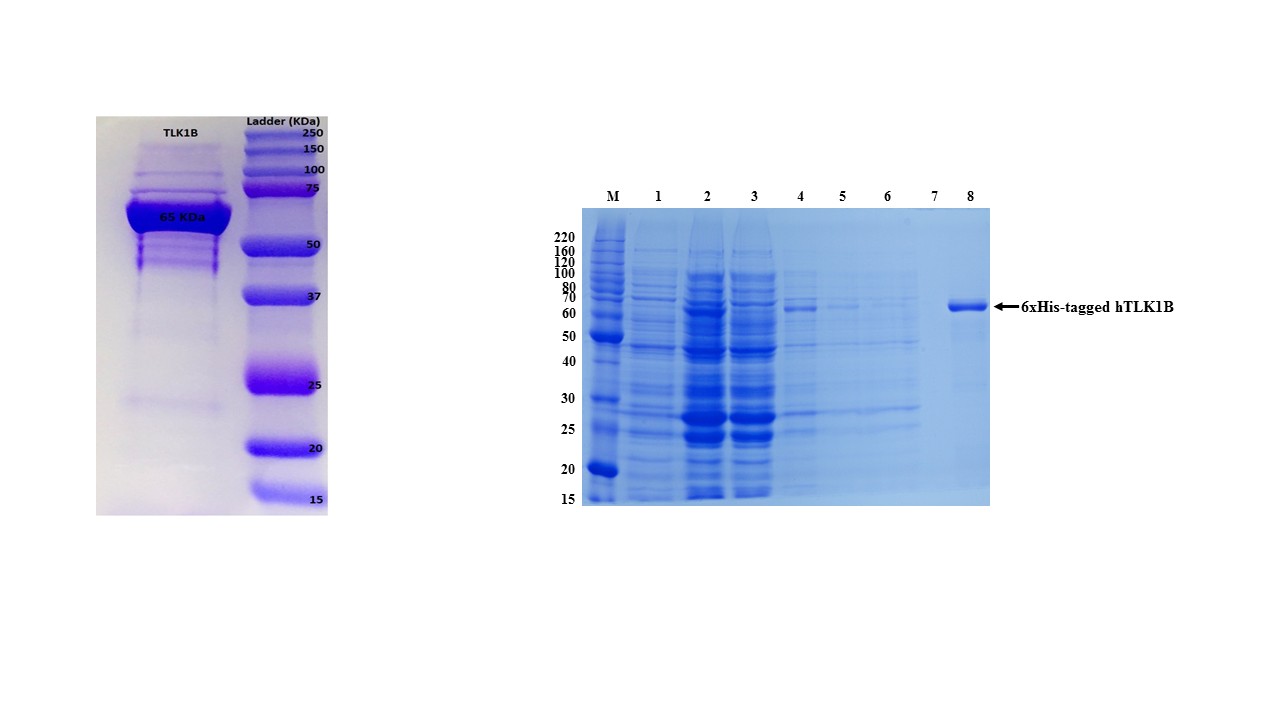
**

**Data 2: Raw data file (LC-MS-MS)**

**m/z S/N Quality Fac. Res. Intens. Area**

420.569 10 1771 3483 3758 675

432.595 8 2612 3675 3108 554

436.643 77 21996 3646 30163 5354

437.604 38 16494 3488 14976 2850

448.640 30 4370 3936 11755 2028

453.594 10 5697 2986 3954 927

459.321 12 11178 745 4574 4157

464.696 232 37615 3816 90022 16666

471.218 13 12719 587 5112 6065

486.972 90 301004 861 34623 29867

506.753 9 837 3152 3336 809

526.731 7 7777 3896 2585 536

554.777 24 9830 4066 9030 1958

556.762 12 9526 3856 4415 1050

578.680 11 5482 3383 4206 1222

606.720 259 45963 4093 97374 24367

609.715 10 4027 2554 3585 1497

618.713 21 4708 4422 7748 1841

628.696 7 5372 2794 2693 1090

634.768 765 268009 3149 285332 101138

635.772 201 17929 4328 74968 17469

644.730 28 58742 1221 10539 9435

655.680 96 68375 1139 35387 36037

656.756 60 3399 5547 22301 4242

666.558 13 2159 1047 4739 5302

668.280 40 31518 1117 14702 14555

672.725 24 11808 4160 8884 2492

696.780 7 2870 3751 2544 868

698.764 6 1648 3361 2251 861

724.817 76 15171 4029 27469 9154

726.804 50 30901 4012 17858 5984

744.934 20 19056 3816 7030 2496

774.725 11 1196 3788 3993 1648

776.749 62 17610 4142 21907 7989

804.802 723 119209 3088 253159 129632

805.790 149 8809 4699 52126 17524

806.802 58 6739 3932 20289 8065

820.821 7 4338 3117 2302 1176

826.783 28 26490 3906 9870 4148

837.809 12 23561 1795 4166 3650

842.750 16 16623 3724 5486 2527

848.759 24 9501 3921 8167 3572

850.813 7 1809 3736 2246 1058

864.717 15 5929 3908 5145 2395

931.033 13 36976 3440 4244 2333

993.483 8 1131 1256 2485 3674

1042.035 63 211657 3770 19720 11863

1098.215 174 244433 3767 52967 34905

1120.174 10 20697 3622 3164 2230

1139.011 6 4514 1792 1890 2900

1155.224 15 12171 3278 4663 3840

1196.235 9 9283 2951 2707 2618

1199.103 29 35718 3677 8498 6571

1207.157 23 170640 3071 6925 6174

1216.125 80 326419 3798 23464 18018

1221.018 12 3847 3668 3395 2818

1244.242 186 414953 3990 54290 41521

1260.243 24 203233 3854 6860 5414

1266.208 12 66391 3770 3377 2736

1301.245 9 16412 3792 2461 2180

1317.173 8 33262 3588 2195 2057

1445.245 13 139835 4254 3688 3441

1510.101 8 4857 3454 2249 2994

1842.472 12 1625 1069 2387 13478

1858.478 27 300053 4062 7817 12247

1909.491 7 23753 4471 2003 3010

1914.453 7 6491 3937 1773 3263

1951.540 8 5978 4077 2038 3740

1993.704 17 54088 3550 4981 9254

2018.656 13 25313 3531 3270 7147

2028.983 12 33263 3059 3361 7301

2064.730 31 5101 4317 9037 13895

2066.739 67 28924 3572 19355 35704

2082.722 12 15628 3374 3217 7297

2123.735 13 20102 3632 3252 7342

2171.773 25 233815 3814 6910 13645

2268.604 7 4999 2617 1664 5401

2283.931 94 744563 4087 25241 50235

2305.837 33 44188 4153 8113 17943

2309.000 9 3809 4547 2216 4492

2321.788 14 3973 4268 3342 7360

2339.033 32 14606 3948 7668 18158

2362.027 6 4804 3715 1582 3841

2392.213 12 5801 4012 2844 6447

2507.250 6 1916 3741 1374 3764

2595.462 25 204891 3849 5791 14908

2611.459 6 1401 3587 1304 3908

2678.760 7 9251 3134 1340 4711

2913.126 54 334448 3910 9950 30507

2970.250 11 74312 3817 2026 6435

3060.667 89 34196 1505 11252 91654

3449.407 24 35908 3247 2230 10424

3515.448 10 880 1007 1130 16583

3670.307 13 3850 858 992 18567

3685.800 286 15888 1943 19219 155734

3700.470 8 926 804 528 10903

3707.824 8 704 1187 379 5615

3742.557 41 28670 2747 2083 14002

3924.159 8 1831 1170 296 4600

3955.662 7 3585 1655 396 4025
